# Supplementary material for: Gauge ambiguities imply Jaynes-Cummings physics remains valid in ultrastrong coupling QED
Source: Nat Commun. 2019 Jan 30;10:499. doi: 10.1038/s41467-018-08101-0 (PMC6354024; doi:10.1038/s41467-018-08101-0)
Supplement: Supplementary file 1 — Supplementary Information [file 41467_2018_8101_MOESM1_ESM.pdf]

Supplementary Information - Gauge ambiguities imply Jaynes-Cummings physics remains valid in ultrastrong coupling QED

Adam Stokes and Ahsan Nazir

Supplementary Note 1: Arbitrary gauge quantisation of the matter-radiation system

In this part our aim is to start from first principles with the Maxwell-Lorentz system of equations and derive a quantum Hamiltonian, that describes the interaction of a material system with a single-mode of radiation. Throughout the derivation we will carefully keep track of the gauge-freedom that is inherent within the electromagnetic potentials. In doing so we arrive at the final result given in the main text wherein the gauge freedom is parametrised by $\alpha \in \mathbb{R}$.

Throughout this section we will frequently use the Helmholtz decomposition of a vector field $\mathbf{V}$ into transverse and longitudinal parts $\mathbf{V}_{\rm T}$ and $\mathbf{V}_{\rm L}$ such that for all $\mathbf{x}$

$$\mathbf{V} = \mathbf{V}_{\rm T} + \mathbf{V}_{\rm L}, \tag{1}$$

$$\nabla \cdot \mathbf{V}_{\rm T}(\mathbf{x}) = 0, \tag{2}$$

$$\nabla \times \mathbf{V}_{\rm L}(\mathbf{x}) = \mathbf{0}. \tag{3}$$

We assume that all vector fields vanish at the boundaries $|\mathbf{x}| \to \infty$, which allows free use of integration by parts such as

$$\int d^3x\, \mathbf{V}(\mathbf{x}) \cdot \nabla f(\mathbf{x}) = -\int d^3x\, f(\mathbf{x}) \nabla \cdot \mathbf{V}(\mathbf{x}). \tag{4}$$

Recalling that $\nabla \times \nabla f(\mathbf{x}) = \mathbf{0}$ for any f and for all $\mathbf{x}$, we have that for any longitudinal field $\mathbf{U}_{\rm L}$ there exists an f such that $\mathbf{U}_{\rm L} = \nabla f$. It follows from Supplementary Eq. (4) that

$$\int d^3x\, \mathbf{V}_{\rm T}(\mathbf{x}) \cdot \mathbf{U}_{\rm L}(\mathbf{x}) = 0 \tag{5}$$

for any vector fields $\mathbf{V}$ and $\mathbf{U}$. These formulae will be frequently used in what follows.

For notational simplicity in the main text we used $\mathbf{A}$ to denote the transverse component of the vector potential. However, here we will deal with both the transverse and full vector potentials. We denote the total vector potential by $\mathbf{A}$ while its gauge-invariant transverse component is denoted $\mathbf{A}_{\rm T}$. The scalar potential is denoted A_0. A gauge transformation of the potentials

$$A_0 \to A_0 - \dot{\chi}, \tag{6}$$

$$\mathbf{A} \to \mathbf{A} + \nabla \chi \tag{7}$$

where χ is arbitrary, leaves the Maxwell-Lorentz equations invariant. By defining $\mathbf{B} = \nabla \times \mathbf{A}$ the non-dynamical Maxwell equation $\nabla \cdot \mathbf{B} = 0$, which specifies the transverasailty of the magnetic field is satisfied identically, because the divergence of the curl is identically zero; $\nabla \cdot \nabla \times \mathbf{V}(\mathbf{x}) = 0$ for any $\mathbf{V}$ and for all $\mathbf{x}$. On the other hand the non-dynamical Gauss law $\nabla \cdot \mathbf{E} = \rho$ where ρ is the charge density, is a primary constraint, which generates gauge transformations, and which indicates redundancy within the unconstrained theory. The remaining constraint required to eliminate this redundancy is a gauge-fixing condition. As will be made precise in what follows, a convenient method of handling the gauge freedom uses the arbitrary transverse component $\mathbf{g}_{\rm T} = \mathbf{g} - \mathbf{g}_{\rm L}$ of the green's function $\mathbf{g}$ for the divergence operator [1, 2]. The green's function is defined by the equation

$$\nabla \cdot \mathbf{g}(\mathbf{x}, \mathbf{x}') = \delta(\mathbf{x} - \mathbf{x}'), \tag{8}$$

but since $\nabla \cdot \mathbf{g}(\mathbf{x}, \mathbf{x}') \equiv \nabla \cdot \mathbf{g}_{\rm L}(\mathbf{x}, \mathbf{x}')$, Supplementary Eq. (8) only fixes $\mathbf{g}_{\rm L} = \mathbf{g} - \mathbf{g}_{\rm T}$ uniquely as

$$\mathbf{g}_{\rm L}(\mathbf{x}, \mathbf{x}') = -\nabla \frac{1}{4\pi |\mathbf{x} - \mathbf{x}'|}. \tag{9}$$

Any field $\mathbf{g}_{\rm T}$ with $\nabla \cdot \mathbf{g}_{\rm T}(\mathbf{x}, \mathbf{x}') = 0$, can be added to $\mathbf{g}_{\rm L}$ in Supplementary Eq. (9) to obtain a $\mathbf{g}$ that satisfies Supplementary Eq. (8).

We consider bound charges $-e$ and $+e$ where the charge $+e$ is stationary and fixed at the origin. For generality we include an additional external potential $V_{\rm ext}(\mathbf{r})$ acting on the charge $-e$ at $\mathbf{r}$. We define the gauge-invariant non-relativistic Lagrangian as [1]

$$L(t) := \frac{1}{2} m \dot{\mathbf{r}}^2 - V_{\rm ext}(\mathbf{r}) - \int d^3x\, (\rho A_0 - \mathbf{J} \cdot \mathbf{A}) + \frac{1}{2} \int d^3x\, \left(\mathbf{E}^2 - \mathbf{B}^2\right) - \frac{d}{dt} \int d^3x\, \mathbf{A} \cdot \mathbf{P} \tag{10}$$

where $\rho(\mathbf{x}) = -e\delta(\mathbf{x}-\mathbf{r}) + e\delta(\mathbf{x})$, $\mathbf{J}(\mathbf{x}) = -e\dot{\mathbf{r}}\delta(\mathbf{x}-\mathbf{r})$ and the polarisation field is defined by $-\nabla\cdot\mathbf{P} = \rho$. Using Supplementary Eq. (8) we obtain

$$\mathbf{P}(\mathbf{x}) := -\int d^3x'\, \mathbf{g}(\mathbf{x},\mathbf{x}')\rho(\mathbf{x}'). \tag{11}$$

Note that $\mathbf{P}_{\rm L}$ is fixed by Supplementary Eq. (9), but $\mathbf{P}_{\rm T}$ is arbitrary. In Supplementary Eq. (10) $\mathbf{E}$ and $\mathbf{B}$ are electric and magnetic fields respectively. For each time t the components of these vector fields belong to the real Hilbert space $L^2(\mathbb{R}^3)$. The final term in Supplementary Eq. (10) is a total time derivative, so it does not affect the equations of motion. It does however ensure that the Lagrangian in Supplementary Eq. (10) is gauge-invariant. The gauge-invariance of $L(t)$ can easily be verified by making a gauge transformation of the potentials, followed by integration by parts, and then use of the continuity equation $\dot{\rho} = -\nabla\cdot\mathbf{J}$.

We identify two constraint functions, which are the Gauss law

$$C_1 := \rho - \nabla\cdot\mathbf{E}, \tag{12}$$

and a constraint on the form of $\mathbf{A}$

$$C_2 := \int d^3x' \mathbf{g}(\mathbf{x}',\mathbf{x})\cdot\mathbf{A}(\mathbf{x}'). \tag{13}$$

From $C_1 = 0$ it follows that $\mathbf{E}_{\rm L} = -\mathbf{P}_{\rm L}$, where $\mathbf{P}_{\rm L}$ is found using Eqs. (11) and (9), while the constraint $C_2 = 0$ is chosen such that the final term in Supplementary Eq. (10), which is a total time derivative, vanishes. It is easily verified using Eqs. (4) and (8) that a set of vector potentials satisfying $C_2 = 0$ identically, are those such that

$$\mathbf{A}(\mathbf{x}) = \mathbf{A}_{\rm T}(\mathbf{x}) + \nabla\int d^3x'\, \mathbf{g}(\mathbf{x}',\mathbf{x})\cdot\mathbf{A}_{\rm T}(\mathbf{x}') \equiv \mathbf{A}_{\rm T}(\mathbf{x}) + \mathbf{A}_{\rm L}(\mathbf{x}) \tag{14}$$

where in writing the second equality we have recalled that the gradient of a function is necessarily longitudinal. Supplementary Eq. (14) shows that we can use the components of the gauge-invariant transverse vector potential $\mathbf{A}_{\rm T}$ to specify any *total* vector potential in the field configuration space that satisfies $C_2 = 0$. A particular choice of $\mathbf{g}_{\rm T}$ uniquely specifies the longitudinal vector potential as

$$\mathbf{A}_{\rm L}(\mathbf{x}) = \nabla\int d^3x'\, \mathbf{g}(\mathbf{x}',\mathbf{x})\cdot\mathbf{A}_{\rm T}(\mathbf{x}') \equiv \nabla\int d^3x'\, \mathbf{g}_{\rm T}(\mathbf{x}',\mathbf{x})\cdot\mathbf{A}_{\rm T}(\mathbf{x}') \tag{15}$$

where we have used Supplementary Eq. (5). It follows from Supplementary Eq. (15) that the longitudinal vector potential is completely independent of $\mathbf{g}_{\rm L}$, and is fully specified by choosing $\mathbf{g}_{\rm T}$. Note that throughout our approach $\mathbf{g}_{\rm T}$ has remained arbitrary. All of the above equations, as well as the constraints $C_1 = 0 = C_2$ hold independently of the choice of $\mathbf{g}_{\rm T}$. Since $\mathbf{g}_{\rm T}$ uniquely specifies $\mathbf{A}_{\rm L}$ the freedom to choose $\mathbf{g}_{\rm T}$ must be interpreted as *gauge* freedom. The only constraint on $\mathbf{g}_{\rm T}$ is transversality, and for reasons of practicality it may also be required that $\mathbf{g}_{\rm T}$ is suitably well-behaved, having for example, a well-defined Fourier transform.

The purpose of encoding gauge-freedom in this way, is that we can now view $\mathbf{A}_{\rm T}$ as the only dynamical coordinate of the electromagnetic field, that is, $\mathbf{A}_{\rm L}$ can be eliminated in favour of $\mathbf{A}_{\rm T}$ and $\mathbf{g}_{\rm T}$ according to Supplementary Eq. (15). In conventional approaches a choice of gauge is made at the outset and the theory is then quantised. In contrast within the present framework quantisation can be achieved while keeping the gauge $\mathbf{A}_{\rm L}$ arbitrary, because it is possible to quantise the theory via Dirac's method without committing to an explicit choice for $\mathbf{g}_{\rm T}$. As detailed below we therefore obtain a fully quantum framework, such that the gauge can be specified at the quantum level by choosing the c-number function $\mathbf{g}_{\rm T}$. Different gauges are then seen to be related by unitary transformations.

Since $\mathbf{A}_{\rm T}$ is the vector potential associated with the Coulomb gauge, we deduce that the scalar potential A_0 accompanying the vector potential $\mathbf{A}$ given in Supplementary Eq. (14) is

$$A_0(\mathbf{x}) = \phi_{\rm coul}(\mathbf{x}) - \frac{\partial}{\partial t}\int d^3x'\, \mathbf{g}(\mathbf{x}',\mathbf{x})\cdot\mathbf{A}_{\rm T}(\mathbf{x}') \tag{16}$$

where $\phi_{\rm coul}$ is, upto a factor of e, the Coulomb potential associated with the charge density ρ. If we substitute Eqs (14) and (16) into Supplementary Eq. (10) and use $C_1 = 0$ we obtain

$$L(t) = L_0(t) - \frac{d}{dt}\chi(t) \tag{17}$$

where $L_0(t)$ is the Lagrangian associated with the Coulomb gauge and is given by [3]

$$L_0(t) = \frac{1}{2}m\dot{\mathbf{r}}^2 - V(\mathbf{r}) + \int d^3x\, \mathbf{J}\cdot\mathbf{A}_{\rm T} + \frac{1}{2}\int d^3x\, \left(\mathbf{E}_{\rm T}^2 - \mathbf{B}^2\right). \tag{18}$$

The arbitrary function $\chi(t)$ appearing in Supplementary Eq. (17) is independent of $\mathbf{g}_{\rm L}$ and is determined entirely through a choice of gauge $\mathbf{g}_{\rm T}$. It is given by

$$\chi(t) = \int d^3x\, \mathbf{A}_{\rm T}\cdot\mathbf{P} \equiv \int d^3x\, \mathbf{A}_{\rm T}\cdot\mathbf{P}_{\rm T}, \tag{19}$$

$$\mathbf{P}_{\rm T}(\mathbf{x}) := -\int d^3x'\, \mathbf{g}_{\rm T}(\mathbf{x},\mathbf{x}')\rho(\mathbf{x}'). \tag{20}$$

In Supplementary Eq. (18) $V(\mathbf{r}) = V_{\rm ext}(\mathbf{r}) + V_{\rm coul}(\mathbf{r})$ with

$$V_{\rm coul}(\mathbf{r}) = \frac{1}{2}\int d^3x\, \rho(\mathbf{x})\phi_{\rm coul}(\mathbf{x}) = \frac{1}{2}\int d^3x\, \mathbf{E}_{\rm L}(\mathbf{x})^2 = \frac{1}{2}\int d^3x\, \mathbf{P}_{\rm L}(\mathbf{x})^2. \tag{21}$$

This term includes the divergent Coulomb self-energy of each charge as well as the inter-charge Coulomb energy. Note that since $L_0(t)$ and $L(t)$ differ by a total time derivative they necessarily yield the same equations of motion.

We can now conveniently parametrise the choice of gauge by restricting our attention to functions $\mathbf{g}_{\rm T}$ that have the form

$$g_{{\rm T},i}(\mathbf{x},\mathbf{x}') := -\alpha\int_0^1 d\lambda\, x_j'\delta_{ij}^{\rm T}(\mathbf{x}-\lambda\mathbf{x}') \tag{22}$$

where $\alpha\in\mathbb{R}$ is arbitrary. When $\alpha=0$ we have $\mathbf{A}=\mathbf{A}_{\rm T}$, which specifies the Coulomb gauge. When $\alpha=1$ Eqs. (11) and (22) yield the well-known multipolar transverse polarisation field in closed form [3]

$$P_{{\rm T},i}(\mathbf{x})|_{\alpha=1} = P_{{\rm mult},i}(\mathbf{x}) = -e\int_0^1 d\lambda\, r_j\delta_{ij}^{\rm T}(\mathbf{x}-\lambda\mathbf{r}). \tag{23}$$

This polarisation field represents a continuum of infinitesimal dipoles each consisting of charges $+e$ and $-e$ that are stacked end-on-end, and which start at the charge $+e$ located at $\mathbf{0}$ and end at the charge $-e$ located at $\mathbf{r}$. The vector potential corresponding to $\alpha=1$ is

$$\mathbf{A}(\mathbf{x}) = \mathbf{A}_{\rm T}(\mathbf{x}) - \nabla\int_0^1 d\lambda\, \mathbf{x}\cdot\mathbf{A}_{\rm T}(\lambda\mathbf{x}), \tag{24}$$

which satisfies $\mathbf{x}\cdot\mathbf{A}(\mathbf{x})=0$. Denoting the Fourier transform of $\mathbf{A}$ by $\tilde{\mathbf{A}}$ we see that $\mathbf{x}\cdot\mathbf{A}(\mathbf{x})=0$ is the position-space version of the condition $\mathbf{k}\cdot\tilde{\mathbf{A}}(\mathbf{k})=0$ that defines the Coulomb gauge. The gauge defined by $\mathbf{x}\cdot\mathbf{A}(\mathbf{x})=0$ is called the *Poincaré* or multipolar gauge [3].

With the restriction given by Supplementary Eq. (22) the Lagrangian in Supplementary Eq. (17) becomes

$$L(t) \equiv L_\alpha(t) = L_0(t) - \frac{d}{dt}\chi_\alpha(t) \tag{25}$$

where

$$\chi_\alpha(t) = \alpha\int d^3x\, \mathbf{A}_{\rm T}\cdot\mathbf{P}_{\rm mult}. \tag{26}$$

Using $L_\alpha(t)$ we can define the following canonical momenta

$$\mathbf{p}_\alpha = \frac{\partial L_\alpha}{\partial\dot{\mathbf{r}}} = m\dot{\mathbf{r}} - e\mathbf{A}_{\rm T}(\mathbf{r}) + e\alpha\nabla\int_0^1 d\lambda\, \mathbf{r}\cdot\mathbf{A}_{\rm T}(\lambda\mathbf{r}), \tag{27}$$

$$\mathbf{\Pi}_{{\rm T},\alpha} = \frac{\delta L_\alpha}{\delta\dot{\mathbf{A}}_{\rm T}} = \dot{\mathbf{A}}_{\rm T} - \alpha\mathbf{P}_{\rm mult} = -\mathbf{E}_{\rm T} - \alpha\mathbf{P}_{\rm mult} \tag{28}$$

where in finding the expression for $\mathbf{p}_\alpha$ we have used

$$-\frac{\partial}{\partial\dot{\mathbf{r}}}\frac{d\chi_\alpha}{dt} = e\alpha\int_0^1 d\lambda\, \left[\mathbf{A}_{\rm T}(\lambda\mathbf{r}) + r_i\nabla A_{{\rm T},i}(\lambda\mathbf{r})\right] = e\alpha\nabla\int_0^1 d\lambda\, \mathbf{r}\cdot\mathbf{A}_{\rm T}(\lambda\mathbf{r}). \tag{29}$$

Here the repeated index is summed and the first equality follows from Supplementary Eq. (26) and the chain rule

$$\frac{d}{dt}A_{\mathrm{T},i}(\lambda\mathbf{r}(t),t)=\dot{A}_{\mathrm{T},i}(\lambda\mathbf{r}(t),t)+\dot{\mathbf{r}}(t)\cdot\nabla A_{\mathrm{T},i}(\lambda\mathbf{r}(t),t). \tag{30}$$

Although we have been able to exhibit expressions for the canonical momenta in an arbitrary gauge α, in order to pass to the canonical formalism we need to determine the algebraic properties of the canonical momenta and the position variables $\mathbf{r}$ and $\mathbf{A}_{\mathrm{T}}$. The Lie algebra of these variables must be consistent with the constraints, and must also suffice to obtain the correct equations of motion once we have obtained the Hamiltonian. Before we commit to the specific form of $\mathbf{g}_{\mathrm{T}}$ given in Supplementary Eq. (22), we will quantise the classical description while keeping $\mathbf{g}_{\mathrm{T}}$ completely arbitrary. This is achieved using Dirac's method [4], which yields the Hamiltonian

$$\begin{aligned}H=&\frac{1}{2m}\left(\mathbf{p}+e\left[\mathbf{A}_{\mathrm{T}}(\mathbf{r})+\nabla\int d^3x\,\mathbf{g}(\mathbf{x},\mathbf{r})\cdot\mathbf{A}_{\mathrm{T}}(\mathbf{x})\right]\right)^2+V(\mathbf{r})\\&+\frac{1}{2}\int d^3x\left[\left(\mathbf{\Pi}_{\mathrm{T}}-\int d^3x'\mathbf{g}_{\mathrm{T}}(\mathbf{x},\mathbf{x}')\rho(\mathbf{x}')\right)^2+(\nabla\times\mathbf{A}_{\mathrm{T}})^2\right]\end{aligned} \tag{31}$$

where the canonical variables $\{\mathbf{r},\mathbf{p},\mathbf{A}_{\mathrm{T}},\mathbf{\Pi}_{\mathrm{T}}\}$ are fully specified by the commutation relations

$$[r_i,p_j]=\mathrm{i}\delta_{ij}, \tag{32}$$
$$[\mathrm{A}_{\mathrm{T,i}}(\mathbf{x}),\Pi_{\mathrm{T},j}(\mathbf{x}')]=\mathrm{i}\delta^{\mathrm{T}}_{ij}(\mathbf{x}-\mathbf{x}'). \tag{33}$$

All other commutators between elements of $\{\mathbf{r},\mathbf{p},\mathbf{A}_{\mathrm{T}},\mathbf{\Pi}_{\mathrm{T}}\}$ vanish identically. All observables are expressed as functions of these operators and Eqs. (32) and (33), and the Hamiltonian in Supplementary Eq. (31) provide all that is needed to obtain the time evolution of a given observable. In particular it is straightforward to verify that the Hamiltonian in Supplementary Eq. (31) yields the correct Maxwell-Lorentz equations. The gauge-invariant vector potential $\mathbf{A}_{\mathrm{T}}$ appearing in Supplementary Eq. (31) belongs to the Coulomb gauge in the sense that $\mathbf{A}\equiv\mathbf{A}_{\mathrm{T}}$ in this gauge, but the Hamiltonian itself has been expressed in an arbitrary gauge g, which is determined by $\mathbf{g}_{\mathrm{T}}$. Using the Heisenberg equation we see that the arbitrary g-gauge canonical momenta $\mathbf{p}$ and $\mathbf{\Pi}_{\mathrm{T}}$ can be identified in terms of the gauge-invariant observables $\{\mathbf{r},\mathbf{A}_{\mathrm{T}},\dot{\mathbf{r}},\dot{\mathbf{A}}_{\mathrm{T}}=-\mathbf{E}_{\mathrm{T}}\}$, and the gauge dependent function $\mathbf{g}_{\mathrm{T}}$, as

$$\mathbf{p}=m\dot{\mathbf{r}}-e\left(\mathbf{A}_{\mathrm{T}}(\mathbf{r})+\nabla\int d^3x\,\mathbf{g}_{\mathrm{T}}(\mathbf{x},\mathbf{r})\cdot\mathbf{A}_{\mathrm{T}}(\mathbf{x})\right)\equiv m\dot{\mathbf{r}}-e\mathbf{A}(\mathbf{r}), \tag{34}$$
$$\mathbf{\Pi}_{\mathrm{T}}(\mathbf{x})=-\mathbf{E}_{\mathrm{T}}(\mathbf{x})-\mathbf{P}_{\mathrm{T}}(\mathbf{x}) \tag{35}$$

where $\mathbf{P}_{\mathrm{T}}$ is determined by $\mathbf{g}_{\mathrm{T}}$ as in Supplementary Eq. (19). This shows clearly that the canonical momenta $\mathbf{p}$ and $\mathbf{\Pi}_{\mathrm{T}}$ are manifestly gauge-dependent. Upon restricting ourselves to the specific form of $\mathbf{g}_{\mathrm{T}}$ given in Supplementary Eq. (22) the canonical momenta in Eqs. (34) and (35) are seen to coincide with those given in Eqs. (27) and (28). Using Eqs. (34) and (35) we see that in *any* gauge H can be written entirely in terms of gauge-invariant observables as the sum of material and field energies;

$$H=H_{\mathrm{matter}}+H_{\mathrm{field}}, \tag{36}$$
$$H_{\mathrm{matter}}:=\frac{1}{2}m\dot{\mathbf{r}}^2+V(\mathbf{r}), \tag{37}$$
$$H_{\mathrm{field}}:=\frac{1}{2}\int d^3x\,\left(\mathbf{E}_{\mathrm{T}}^2+\mathbf{B}^2\right). \tag{38}$$

A unitary gauge-fixing transformation between gauges $\mathbf{g}_{\mathrm{T}}$ and $\mathbf{g}'_{\mathrm{T}}$ can be defined as

$$R_{gg'}:=\exp\left[\mathrm{i}\int d^3\mathbf{x}\,[\mathbf{P}_{\mathrm{T},g'}(\mathbf{x})-\mathbf{P}_{\mathrm{T},g}(\mathbf{x})]\cdot\mathbf{A}_{\mathrm{T}}(\mathbf{x})\right] \tag{39}$$

where $\mathbf{P}_{\mathrm{T},g}$ and $\mathbf{P}_{\mathrm{T},g'}$ are defined as in Supplementary Eq. (19) in terms of $\mathbf{g}_{\mathrm{T}}$ and $\mathbf{g}'_{\mathrm{T}}$ respectively. When used to transform the canonical momenta in Eqs. (34) and (35) $R_{gg'}$ replaces the function $\mathbf{g}_{\mathrm{T}}$ with the alternative choice $\mathbf{g}'_{\mathrm{T}}$.

Let us now return to the specific form of $\mathbf{g}_{\mathrm{T}}$ given in Supplementary Eq. (22), wherein the freedom to choose a gauge reduces to the freedom to choose the value of the real parameter α. In terms of this form of $\mathbf{g}_{\mathrm{T}}$ the canonical momenta and Hamiltonian are found using Eqs. (34) and (35), and Supplementary Eq. (31). As noted previously, in this case the canonical momenta are seen to coincide with those given in Supplementary Eq. (27). The unitary

gauge-fixing transformation between different gauges α and α' takes the form of a generalised Power-Zienau-Woolley transformation;

$$R_{\alpha\alpha'} := \exp\left[\mathrm{i}(\alpha-\alpha')\int d^3\mathbf{x}\,\mathbf{P}_{\rm mult}(\mathbf{x})\cdot\mathbf{A}_{\rm T}(\mathbf{x})\right]. \tag{40}$$

The usual Power-Zienau-Woolley transformation, which is used to relate the Coulomb and Poincaré gauges is obtained if $\alpha-\alpha'=1$. The α-gauge polarisation field is $\alpha\mathbf{P}_{\rm mult}$. A multipole expansion of the polarisation field allows one to perform the electric-dipole approximation as $P^{\rm EDA}_{{\rm mult},i} = -er_i\delta^{\rm T}_{ij}(\mathbf{x})$. Equivalently, the dipole approximation can be realised via

$$g^{\rm EDA}_{{\rm T},i}(\mathbf{x},\mathbf{x}') = -\alpha x'_j\delta^{\rm T}_{ij}(\mathbf{x}). \tag{41}$$

The dipole approximated α-gauge canonical momenta can then be read-off from Eqs. (34) and (35) as

$$\mathbf{p}_\alpha = m\dot{\mathbf{r}} - e(1-\alpha)\mathbf{A}_{\rm T}(\mathbf{0}), \tag{42}$$

$$\Pi_{{\rm T}.\alpha,i}(\mathbf{x}) = -E_{{\rm T},i}(\mathbf{x}) - \alpha d_j\delta^{\rm T}_{ij}(\mathbf{x}) \tag{43}$$

where $\mathbf{d}=-e\mathbf{r}$. The unitary gauge-fixing transformation becomes

$$R_{\alpha\alpha'} := \exp\left[\mathrm{i}(\alpha-\alpha')\mathbf{d}\cdot\mathbf{A}_{\rm T}(\mathbf{0})\right]. \tag{44}$$

Since the gauge-fixing transformation remains unitary the dipole approximation does not destroy the gauge-invariance of the theory. The dipole approximated Hamiltonian is

$$\begin{aligned} H =& \frac{1}{2m}[\mathbf{p}_\alpha + e(1-\alpha)\mathbf{A}_{\rm T}(\mathbf{0})]^2 + V(\mathbf{r}) + \frac{1}{2}\int d^3x\left(\left[\Pi_{{\rm T},\alpha,i}(\mathbf{x}) + \alpha d_j\delta^{\rm T}_{ij}(\mathbf{x})\right]^2 + [\nabla\times\mathbf{A}_{\rm T}(\mathbf{x})]^2\right) \\ =& \frac{1}{2m}[\mathbf{p}_\alpha + e(1-\alpha)\mathbf{A}_{\rm T}(\mathbf{0})]^2 + V(\mathbf{r}) + \frac{1}{2}\int d^3k\left(\left|\tilde{\mathbf{\Pi}}_{{\rm T},\alpha}(\mathbf{k}) + \frac{\alpha}{(2\pi)^3}\sum_\lambda \boldsymbol{\varepsilon}_\lambda(\mathbf{k})[\mathbf{d}\cdot\boldsymbol{\varepsilon}_\lambda(\mathbf{k})]\right|^2 + |\mathrm{i}\mathbf{k}\times\tilde{\mathbf{A}}_{\rm T}(\mathbf{k})|^2\right) \end{aligned} \tag{45}$$

where $\boldsymbol{\varepsilon}_\lambda(\mathbf{k}),\ \lambda=1,2$ are mutually orthogonal unit polarisation vectors that are both orthogonal to $\mathbf{k}$, and $\tilde{f}$ denotes the Fourier transform of f. We have also used

$$P^{\rm EDA}_{{\rm mult},i}(\mathbf{x}) = d_j\delta^{\rm T}_{ij}(\mathbf{x}) = \int\frac{d^3k}{(2\pi)^3}\sum_\lambda \varepsilon_{\lambda,i}(\mathbf{k})[\mathbf{d}\cdot\boldsymbol{\varepsilon}_\lambda(\mathbf{k})]e^{\mathrm{i}\mathbf{k}\cdot\mathbf{x}}. \tag{46}$$

The above expressions are applicable for general field operators $\mathbf{A}_{\rm T}$ and $\mathbf{\Pi}_{{\rm T},\alpha}$. We define the operator

$$a_{\alpha,\lambda}(\mathbf{k}) := \sqrt{\frac{1}{2\omega}}\left(\omega\tilde{A}_{{\rm T},\lambda}(\mathbf{k}) + \mathrm{i}\tilde{\Pi}_{{\rm T},\alpha,\lambda}(\mathbf{k})\right) \tag{47}$$

where $\tilde{A}_{{\rm T},\lambda}(\mathbf{k}) = \boldsymbol{\varepsilon}_\lambda(\mathbf{k})\cdot\tilde{\mathbf{A}}_{\rm T}(\mathbf{k})$ and $\tilde{\Pi}'_{{\rm T},\alpha,\lambda}(\mathbf{k}) = \boldsymbol{\varepsilon}_\lambda(\mathbf{k})\cdot\tilde{\mathbf{\Pi}}'_{{\rm T},\alpha}(\mathbf{k})$. From the transverse canonical commutation relation

$$[A_{{\rm T},i}(\mathbf{x}),\Pi_{{\rm T},\alpha,j}(\mathbf{x}')] = \mathrm{i}\delta^{\rm T}_{ij}(\mathbf{x}-\mathbf{x}') \tag{48}$$

it follows that

$$[a_{\alpha,\lambda}(\mathbf{k}),a^\dagger_{\alpha,\lambda'}(\mathbf{k}')] = \delta_{\lambda\lambda'}\delta(\mathbf{k}-\mathbf{k}'). \tag{49}$$

The operators $a_{\alpha,\lambda}(\mathbf{k})$ and $a^\dagger_{\alpha,\lambda}(\mathbf{k})$ are recognisable as annihilation and creation operators for a photon with momentum $\mathbf{k}$ and polarisation λ. In terms of these operators the canonical fields support the Fourier representations

$$\begin{aligned} \mathbf{A}_{\rm T}(\mathbf{x}) &= \int d^3k\sum_\lambda g\boldsymbol{\varepsilon}_\lambda(\mathbf{k})\left(a^\dagger_{\alpha,\lambda}(\mathbf{k})e^{-\mathrm{i}\mathbf{k}\cdot\mathbf{x}} + a_{\alpha,\lambda}(\mathbf{k})e^{\mathrm{i}\mathbf{k}\cdot\mathbf{x}}\right), \\ \mathbf{\Pi}_{{\rm T},\alpha}(\mathbf{x}) &= \mathrm{i}\int d^3k\sum_\lambda \omega g\boldsymbol{\varepsilon}_\lambda(\mathbf{k})\left(a^\dagger_{\alpha,\lambda}(\mathbf{k})e^{-\mathrm{i}\mathbf{k}\cdot\mathbf{x}} - a_{\alpha,\lambda}(\mathbf{k})e^{i\mathbf{k}\cdot\mathbf{x}}\right) \end{aligned} \tag{50}$$

where $\omega=|\mathbf{k}|$ and $g := 1/\sqrt{2\omega(2\pi)^3}$.

If we assume an implicit cavity with volume v that satisfies periodic boundary conditions, the continuous label $\mathbf{k}$ becomes discrete. The pair $\mathbf{k}\lambda$ then labels a radiation mode. As a less realistic, but simpler model for the cavity we may restrict our attention to a single mode, in which case the field operators become

$$\mathbf{A}_{\rm T} = g\boldsymbol{\varepsilon}\left(a_\alpha^\dagger + a_\alpha\right), \tag{51}$$

$$\mathbf{\Pi}_{{\rm T},\alpha} = {\rm i}\omega g\boldsymbol{\varepsilon}\left(a_\alpha^\dagger - a_\alpha\right), \tag{52}$$

where $[a_\alpha, a_\alpha^\dagger] = 1$ and $g = 1/\sqrt{2\omega v}$. Eqs. (51) imply that the cavity canonical operators now satisfy the commutation relation

$$[A_{{\rm T},i}, \Pi_{{\rm T},\alpha,j}] = \frac{{\rm i}\varepsilon_i\varepsilon_j}{v} \tag{53}$$

as specified in the main text. For consistency with Eqs. (34), (35), (42), and (43), within the single-mode approximation we must also restrict the Fourier transform of the polarisation field $\mathbf{P}_{\rm mult}$ in Supplementary Eq. (46) to a single mode such that the transverse electric field satisfies $\mathbf{E}_{\rm T} = -\dot{\mathbf{A}}_{\rm T} = -\mathbf{\Pi}_{{\rm T},\alpha} - \alpha\mathbf{P}_{\rm mult}$. If in the single-mode approximation we write the Hamiltonian in Supplementary Eq. (45) as

$$H = \frac{1}{2m}(\mathbf{p}_\alpha + e(1-\alpha)\mathbf{A}_{\rm T})^2 + V(\mathbf{r}) + \frac{v}{2}\left(\left[\mathbf{\Pi}_{{\rm T},\alpha} + \frac{\alpha\boldsymbol{\varepsilon}(\mathbf{d}\cdot\boldsymbol{\varepsilon})}{v}\right]^2 + \omega^2\mathbf{A}_{\rm T}^2\right) \tag{54}$$

where we have restricted the polarisation field to a single polarisation as $\alpha\mathbf{P}_{\rm mult} = \alpha\boldsymbol{\varepsilon}(\mathbf{d}\cdot\boldsymbol{\varepsilon})/v$, we obtain

$$\mathbf{E}_{\rm T} = -\dot{\mathbf{A}}_{\rm T} = -g\boldsymbol{\varepsilon}\left(\dot a_\alpha^\dagger + \dot a_\alpha\right) = -{\rm i}\omega g\boldsymbol{\varepsilon}\left(a_\alpha^\dagger - a_\alpha\right) - \frac{\alpha\boldsymbol{\varepsilon}(\mathbf{d}\cdot\boldsymbol{\varepsilon})}{v} = -\mathbf{\Pi}_{\rm T} - \alpha\mathbf{P}_{\rm mult} \tag{55}$$

as required. We therefore obtain a consistent single-mode theory with Hamiltonian given by Supplementary Eq. (54), and cavity canonical operators $\mathbf{A}_{\rm T}$ and $\mathbf{\Pi}_{{\rm T},\alpha}$ fully specified by Eqs. (51). Like the dipole approximation the single-mode approximation preserves the gauge-invariance of the theory, because it does not alter the unitary property of the gauge-fixing transformation $R_{\alpha\alpha'}$, which retains the form given in Supplementary Eq. (44) but with $\mathbf{A}_{\rm T}$ specifying the single-mode vector potential from Supplementary Eq. (51). The Hamiltonian and the Heisenberg equation yield

$$m\dot{\mathbf{r}} = \mathbf{p}_\alpha + e(1-\alpha)\mathbf{A}_{\rm T}, \tag{56}$$

$$\mathbf{E}_{\rm T} = -\mathbf{\Pi}_{{\rm T},\alpha} - \frac{\alpha\boldsymbol{\varepsilon}(\mathbf{d}\cdot\boldsymbol{\varepsilon})}{v} \tag{57}$$

which are the single-mode versions of Eqs. (42) and (43). Eqs. (56) and (57) allow us to write the Hamiltonian as $H = E_{\rm matter} + E_{\rm cavity}$ where $E_{\rm matter} = m\dot{\mathbf{r}}^2/2 + V(\mathbf{r})$ and $E_{\rm cavity} = v(\mathbf{E}_{\rm T}^2 + \omega^2\mathbf{A}_{\rm T}^2)/2$. This is merely the dipole-approximated single-mode version of Supplementary Eq. (36).

In summary, the restriction to functions $\mathbf{g}_{\rm T}$ of the form given in Supplementary Eq. (22), together with the electric-dipole approximation, and the restriction to a single-mode of radiation yield the expressions given in the main text. For simplicity, in the main text we use the notation $\mathbf{A}$ for $\mathbf{A}_{\rm T}$ and $\mathbf{\Pi}_\alpha$ for $\mathbf{\Pi}_{{\rm T},\alpha}$. The gauge is completely determined by α. The theory is gauge-invariant in the sense that the predictions concerning any gauge-invariant observable can be calculated using any gauge and these predictions are unique. Choosing a specific gauge is merely a matter of convenience for performing calculations. As explained in the main text this is no longer the case within two-level models for the material system.

Supplementary Note 2: Can gauge-invariant predictions be obtained from two-level models in the weak-coupling regime?

A well-known drawback of two-level models is the breakdown of sum-rules involving matrix elements of operators which satisfy the CCR algebra. This occurs because the CCR algebra cannot be supported by a finite-dimensional Hilbert space. A well-known example is given by the Thomas-Reiche-Kuhn (TRK) sum rule [5]

$$\sum_{r=0}^{\infty}\epsilon_{rs}d_{rs}^i d_{sr}^j = \frac{e^2}{2m}\delta_{ij} \tag{58}$$

where $d_{rs}^i = \langle\epsilon_\alpha^r| - er_i|\epsilon_\alpha^s\rangle$ and $\epsilon_{rs} = \epsilon_r - \epsilon_s$. In the full (infinite-dimensional) atomic Hilbert space the right-hand-side of this identity is independent of the dipole level s. Yet, when considering a two-level dipole the values of the indices

r and s on the right-hand-side of Supplementary Eq. (58) must be 0 or 1. For the ground state $\sigma_\alpha^-\sigma_\alpha^+$ with $s=0$ Supplementary Eq. (58) becomes $\omega_{\rm m}({\bf d}\cdot\boldsymbol{\varepsilon})^2 = e^2/2m$ while for the excited state $\sigma_\alpha^+\sigma_\alpha^-$ with $s=1$ Supplementary Eq. (58) becomes $\omega_{\rm m}({\bf d}\cdot\boldsymbol{\varepsilon})^2 = -e^2/2m$. These relations cannot be simultaneously satisfied. Furthermore, the second relation implies that $m<0$.

In conventional atomic physics it is necessary to use the TRK sum rule (58) in order to show invariance, between the Coulomb and Poincaré gauges, of the Lamb shift derived using stationary second order perurbation theory [6, 7]. Thus, one should already anticipate difficulties in the maintenance of gauge-invariance in two-level models even within the conventional weak-coupling regime. We note that the TRK sum rule has also been used extensively in the strong and ultrastrong light-matter physics literature, but in a different context. There it is applied on the level of the infinite-dimensional atom with the aim of deriving inequalities for atomic transitions involving the lowest two levels.

Our motivation here is different; we are concerned with the question of whether it is possible to establish gauge-invariance of predictions using non-equivalent two-level models. We therefore consider whether or not it is possible to elicit gauge-invariance of level-shifts through any systematic application of the TRK sum rule, *after* the two-level approximation has been made in the arbitrary α-gauge. We show that provided the TRK sum rule is applied judiciously, the precise meaning of which will be specified below, then gauge-invariance (α-independence) can be elicited for the energy levels calculated in different two-level models, but only upto second order in e. Thus, the main conclusion of this Supplementary Note is that even with a somewhat ad hoc application of the TRK sum rule, in any two-level model one can at best expect to obtain α-independent predictions upto order d^2 only. At the end of this section we also briefly discuss alternative definitions of two-level models.

We consider the two-level model Hamiltonian H_2^α given in Eq. (5) of the main text, second order perturbation theory and judicious use of the relations $\omega_{\rm m}({\bf d}\cdot\boldsymbol{\varepsilon})^2 = e^2/2m$ and $\omega_{\rm m}({\bf d}\cdot\boldsymbol{\varepsilon})^2 = -e^2/2m$ yield α-independent expressions for the ground and first excited energies. The ground energy of H_2^α found using second order perturbation theory in the interaction Hamiltonian is

$$\langle G_2^\alpha|\, H_2^\alpha\, |G_2^\alpha\rangle \approx \frac{\omega_\alpha}{2} + \Delta_\alpha - \frac{{u_\alpha^+}^2}{\omega_{\rm m}+\omega_\alpha}. \tag{59}$$

Upon use of $\omega_{\rm m}({\bf d}\cdot\boldsymbol{\varepsilon})^2 = e^2/2m$ appropriate for the ground state we obtain to order d^2 the α-independent result

$$\langle G_2^\alpha|\, H_2^\alpha\, |G_2^\alpha\rangle \approx \epsilon_0 + \frac{\omega}{2} + [g{\bf d}\cdot\boldsymbol{\varepsilon}]^2\frac{\omega_{\rm m}\omega}{\omega_{\rm m}+\omega}. \tag{60}$$

Similarly, the first excited energy of H_2^α is to order d^2 given by

$$\langle E_2^\alpha|\, H_2^\alpha\, |E_2^\alpha\rangle \approx \frac{\omega_\alpha}{2} + \Delta_\alpha + \frac{{u_\alpha^-}^2}{\omega_{\rm m}-\omega_\alpha} \approx \epsilon_0 + \frac{\omega}{2} + [g{\bf d}\cdot\boldsymbol{\varepsilon}]^2\frac{\omega_{\rm m}\omega}{\omega_{\rm m}-\omega}. \tag{61}$$

where in writing the second equality we have used $\omega_{\rm m}({\bf d}\cdot\boldsymbol{\varepsilon})^2 = -e^2/2m$ appropriate for the excited state. As in the case of an infinite-dimensional atom, the above exhibition of gauge-invariance relies upon the elimination of the mass m in favour of the dipole moment $\bf d$ and other parameters [6, 7]. The difference in the two-level model case is only that the separate relations $\omega_{\rm m}({\bf d}\cdot\boldsymbol{\varepsilon})^2 = e^2/2m$ and $\omega_{\rm m}({\bf d}\cdot\boldsymbol{\varepsilon})^2 = -e^2/2m$ must be used for the ground and excited states respectively, which essentially accounts for the modification of the operator algebra incurred by the two-level truncation [7].

Motivated by the above derivations we now show that the bare mass can similarly be eliminated on the level of the Hamiltonian, and this yields another possible form of two-level model. More precisely, if within the two-level truncation we apply the appropriate relations $\omega_{\rm m}({\bf d}\cdot\boldsymbol{\varepsilon})^2 = e^2/2m$ for the ground state and $\omega_{\rm m}({\bf d}\cdot\boldsymbol{\varepsilon})^2 = -e^2/2m$ for the excited state in the self-energy term $e^2(1-\alpha)^2{\bf A}^2/2m$, then this term becomes $-\omega_{\rm m}({\bf d}\cdot\boldsymbol{\varepsilon})^2(1-\alpha)^2\sigma_\alpha^z{\bf A}^2$ where $\sigma_\alpha^z = [\sigma_\alpha^+,\sigma_\alpha^-]$. All dependence on the bare mass m has now been eliminated and we obtain a well-defined two-level model Hamiltonian given by

$$\bar H_2^\alpha = \omega_{\rm m}\sigma_\alpha^+\sigma_\alpha^- + \Delta_\alpha + \omega_{\rm m}(1-\alpha)({\bf d}\cdot{\bf A})\sigma_\alpha^y + \alpha({\bf d}\cdot{\bf \Pi}_\alpha)\sigma_\alpha^x - \omega_{\rm m}({\bf d}\cdot\boldsymbol{\varepsilon})^2(1-\alpha)^2\sigma_\alpha^z{\bf A}^2 + \omega\left(a_\alpha^\dagger a_\alpha + \frac{1}{2}\right) \tag{62}$$

where $\sigma_\alpha^y = {\rm i}(\sigma_\alpha^- - \sigma_\alpha^+)$ and $\sigma_\alpha^x = \sigma_\alpha^+ + \sigma_\alpha^-$.

In contrast to H_2^α a simple approximate relation can be given between the average energy found using distinct two-level model Hamiltonians $\bar H_2^\alpha$ and $\bar H_2^{\alpha'}$ when $\alpha\neq\alpha'$. To see this we note that the gauge transformation $R_{0\alpha} = {\rm e}^{{\rm i}e\alpha{\bf r}\cdot{\bf A}}$ is a function of the canonical variables ${\bf r},\ {\bf A}\in{\bf y}_0$. We express this functional dependence as $R_{0\alpha}\equiv R_{0\alpha}({\bf y})$ where ${\bf y}\equiv{\bf y}_0$, and we define the unitary operator $U_\alpha = R_{0\alpha}(P^0{\bf y}P^0) = \cos(\alpha{\bf d}\cdot{\bf A}) - {\rm i}\sigma^x\sin(\alpha{\bf d}\cdot{\bf A})$ where $\sigma^k\equiv\sigma_0^k,\ k=\pm,x,y,z$. Noting further that $\bar H_2^0$ is a function of the Coulomb-gauge Rabi model raising and lowering operators, expressed

as $\bar{H}_2^0(\sigma^\pm)$, it is straightforward to show using this notation that $U_\alpha \bar{H}_2^0(\sigma^\pm)U_\alpha^{-1} \approx \bar{H}_2^\alpha(\sigma^\pm)$ where the approximate equality means that equality holds upto second order in η. If in the Coulomb gauge two-level model with Hamiltonian $\bar{H}_2^0$ we represent an arbitrary state $\mathcal{S}$ by $|\psi^0\rangle = \sum_n \psi_n |\bar{E}_{2,n}^0\rangle$, then in the α-gauge two-level model with Hamiltonian $\bar{H}_2^\alpha$, $\mathcal{S}$ is represented by $|\psi^\alpha\rangle = \sum_n \psi_n |\bar{E}_{2,n}^\alpha\rangle \approx U_\alpha |\psi^0\rangle$ and we therefore obtain $\langle\psi^0| \bar{H}_2^0 |\psi^0\rangle \approx \langle\psi^\alpha| \bar{H}_2^\alpha |\psi^\alpha\rangle$.

Finally we remark on the possibility of yet another form of two-level model. In the main text the two-level model Hamiltonian is found by projecting the canonical operators $\mathbf{y}_\alpha$ as $P^\alpha \mathbf{y}_\alpha P^\alpha$ and substituting the projected operators into the interaction Hamiltonian. This is only equivalent to a projection of the Hamiltonian itself $P^\alpha H P^\alpha$ if the interaction is a linear function of the material operators within $\mathbf{y}_\alpha$. In the non-truncated Hamiltonian of Eqs. (9), (10) and (11) of the main text, however there is a non-linear term $\alpha^2/2v(\boldsymbol{\varepsilon}\cdot\mathbf{d})^2$. The lowest two energy levels obtained for two-level models of the form $P^\alpha H P^\alpha$ are therefore quantitatively different in some cases to those found in the main text. However the main conclusions remain unchanged. As stated within the main text a comprehensive comparison of distinct types of two-level model is beyond the scope of this article and will be given elsewhere.

Supplementary Note 3: Fluxonium LC-oscillator Hamiltonian

Here we derive the full Hamiltonian describing a fluxonium-LC oscillator circuit. The fluxonium is described by flux operator ϕ with conjugate momentum ξ such that $[\phi, \xi] = \mathrm{i}$. The Hamiltonian is [8]

$$H_\mathrm{m} = \frac{E_\mathrm{c}}{e^2}\xi^2 - E_\mathrm{J}\cos\left(2e[\phi - \phi_\mathrm{ext}]\right) + 2e^2 E_\mathrm{l}\phi^2 \tag{63}$$

where E_c, E_J and E_l are the capacitive, Josephson and inductive energies respectively, and ϕ_ext is the applied external flux. The LC-oscillator is described by flux operator θ with conjugate momentum ζ such that $[\theta, \zeta] = \mathrm{i}$. Its Hamiltonian is

$$H_{LC} = \frac{\zeta^2}{2C} + \frac{\theta^2}{2L} \tag{64}$$

where C and L are the capacitance and inductance respectively.

There is considerable freedom in describing the coupling between the fluxonium and the oscillator. Capacitively coupling the systems is achieved through the replacement $\xi \to \xi + \zeta$. This can be viewed as analogous to the replacement $\mathbf{p} \to \mathbf{p} + e\mathbf{A}$, which results in the Coulomb-gauge coupling between an atom and a cavity. Inductively coupling the fluxonium and oscillator is achieved through the replacement $\theta \to \theta + \phi$, which can be viewed as analogous to the replacement $\mathbf{\Pi} \to \mathbf{\Pi} + \boldsymbol{\epsilon}(\boldsymbol{\epsilon}\cdot\mathbf{d})/v$ that gives the Poincaré-gauge coupling between an atom and a cavity. We have already seen in the context of an atom-cavity system that this freedom in the description of the coupling is a gauge-freedom, and that the different descriptions are unitarily related. Analogously we call the capacitive coupling the charge-gauge description, and we call the inductive coupling the flux-gauge description. Making the replacement $\xi \to \xi + \zeta$ in the Hamiltonian in Supplementary Eq. (63) and adding the bare oscillator Hamiltonian in Supplementary Eq. (64) yields the Hamiltonian expressed in the charge-gauge;

$$H = \frac{E_\mathrm{c}}{e^2}(\xi + \zeta)^2 - E_\mathrm{J}\cos\left(2e[\phi - \phi_\mathrm{ext}]\right) + 2e^2 E_\mathrm{l}\phi^2 + \frac{\zeta^2}{2C} + \frac{\theta^2}{2L}. \tag{65}$$

If we define the unitary gauge transformation $R_{01} = \mathrm{e}^{\mathrm{i}\phi\zeta}$ then we can define new canonical operators $\xi_1 = R_{01}^{-1}\xi R_{01}$ and $\theta_1 = R_{01}^{-1}\theta R_{01}$ in terms of which the Hamiltonian is expressed in the flux-gauge;

$$H = \frac{E_\mathrm{c}}{e^2}\xi_1^2 - E_\mathrm{J}\cos\left(2e[\phi - \phi_\mathrm{ext}]\right) + 2e^2 E_\mathrm{l}\phi^2 + \frac{\zeta^2}{2C} + \frac{1}{2L}(\theta_1 + \phi)^2. \tag{66}$$

More generally, we define the unitary gauge transformation $R_{0\alpha} = \mathrm{e}^{i\alpha\phi\zeta}$ and associated α-gauge canonical operators by $\xi_\alpha = R_{0\alpha}^{-1}\xi R_{0\alpha}$ and $\theta_\alpha = R_{0\alpha}^{-1}\theta R_{0\alpha}$. The Hamiltonian expressed in terms of the α-gauge canonical operators $\mathbf{y}_\alpha = \{\phi,\ \xi_\alpha,\ \theta_\alpha,\ \zeta\}$ is

$$H = \frac{E_\mathrm{c}}{e^2}[\xi_\alpha + (1-\alpha)\zeta]^2 + 2e^2 E_\mathrm{l}\phi^2 - E_\mathrm{J}\cos\left(2e[\phi - \phi_\mathrm{ext}]\right) + \frac{\zeta^2}{2C} + \frac{1}{2L}[\theta_\alpha + \alpha\phi]^2. \tag{67}$$

In all gauges the canonical operators satisfy the canonical commutation relations due to the unitarity of the gauge transformation. The Hamiltonian H has α-independent (gauge-invariant) spectrum. More generally, the predictions for any observable $O = o^\alpha(\mathbf{y}_\alpha) = o^{\alpha'}(\mathbf{y}_{\alpha'})$ can be calculated using any gauge and these predictions are unique. The

charge and flux-gauge descriptions are obtained by choosing $\alpha = 0$ and $\alpha = 1$ respectively. The α-gauge two-level model is obtained in exact analogy with the atom-cavity formalism by using the projection P^α onto the first two eigenstates of the α-gauge bare fluxonium Hamiltonian

$$H_{\rm m}^\alpha = \frac{E_{\rm c}}{e^2}\xi_\alpha^2 - E_{\rm J}\cos\left(2e[\phi - \phi_{\rm ext}]\right) + 2e^2 E_{\rm l}\phi^2. \tag{68}$$

Supplementary Note 4: Further anaysis via an effective Hamiltonian in the dispersive regime

Here we provide more detailed analysis of predictions in different gauges with the aim of understanding which two-level models will be more accurate in which regimes. Our results are derived using Schrieffer-Wolff perturbation theory (also known as Van Vleck perturbation theory), and our presentation is similar to the one in Ref. [9]. An effective Hamiltonian describing a linearly coupled material-oscillator system is derived, which is valid within the dispersive regime $|\epsilon_{nm} - \omega| \gg |g_{nm}|\sqrt{N+1}$ where g_{nm} are the coupling constants of the linear interaction, ϵ_{nm} are the material transition frequencies and N denotes photon population. For the fluxonium-LC system we consider, this regime requires detunings δ sufficiently far from resonance in comparison to the coupling strengths η even if $N = 0$. Although this is not always the case in the regimes we consider, the Schrieffer-Wolff perturbation method can be used to gain physical insight into the regimes of large and small δ for various coupling strengths, and can thereby reveal why particular two-level models become more accurate in particular regimes. The method uses an appropriate unitary transformation e^{iS} and perturbation theory to derive a diagonal Hamiltonian describing the system.

As in Ref. [9] let us consider the general linear-coupling of a multi-level material system and a single oscillator. Partitioned into unperturbed and interaction parts the most general such Hamiltonian is

$$H = H_0 + V \equiv \left[\sum_n \epsilon_n |\epsilon_n\rangle\langle\epsilon_n| + \Omega a^\dagger a\right] + \left[\sum_{nm} g_{nm}|\epsilon_n\rangle\langle\epsilon_m| a + {\rm H.c.}\right]. \tag{69}$$

If in the fluxonium-LC Hamiltonian of Eq. (6) of the main text we ignore the flux self-energy contribution $\alpha^2\phi^2/2L$, which is not important for our analysis, then the Hamiltonian in Eq. (6) of the main text is of the form of H in Supplementary Eq. (69) upto a c-number offset term.

Using an appropriate unitary transformation e^{iS} and second order perturbation theory, in the dispersive regime an effective Hamiltonian can be derived from Supplementary Eq. (69) as [9]

$$H_{\rm eff} = \omega {\rm a}^\dagger {\rm a} + \sum_n (\epsilon_n + \kappa_n)|n\rangle\langle n| + \sum_n \chi_n {\rm a}^\dagger {\rm a}|n\rangle\langle n| \tag{70}$$

where $|n\rangle\langle n| = e^{iS}|\epsilon_n\rangle\langle\epsilon_n|e^{-iS}$ and ${\rm a} = e^{iS}ae^{-iS}$ are dressed operators. For details of the method of derivation of $H_{\rm eff}$ we refer to [9]. The additional energy coefficients κ_n and χ_n are given by ordinary stationary second order perturbation theory and are found to be [9]

$$\kappa_n = \sum_m \frac{|g_{nm}|^2}{\epsilon_{nm} - \Omega}, \qquad \chi_n = 2\sum_m |g_{nm}|^2 \frac{\epsilon_{nm}}{\epsilon_{nm}^2 - \Omega^2}. \tag{71}$$

The κ_n are material level shifts (Lamb-shifts) while the χ_n are ac-Stark shifts, which can be understood as material level-dependent oscillator shifts $\omega \to \omega + \chi_n$, or as oscillator-dependent material shifts; $\epsilon_n \to \epsilon_n + \chi_n a^\dagger a$. It is important to note that for the fluxonium-LC Hamiltonian in Eq. (6) of the main text the unperturbed Hamiltonian H_0 as well as the interaction V are different in each gauge. More precisely, for H in Supplementary Eq. (69) to match the fluxonium-LC Hamiltonian in Eq. (6) of the main text the oscillator frequency Ω must be identified as ω_α and the coupling constants g_{nm} must be identified as

$$g_{nm} = \frac{\phi_{nm}}{\sqrt{2\omega_\alpha L}}(\epsilon_{nm}(1-\alpha) + \omega_\alpha\alpha). \tag{72}$$

In the general α-gauge we obtain κ_n and χ_n through substitution of $\Omega = \omega_\alpha$ and Supplementary Eq. (72) into Supplementary Eq. (71). The expressions obtained are α-dependent. Flux-gauge coupling is weighted in Supplementary Eq. (72) by α and depends on the matrix elements ϕ_{nm}, which are shown for the first few fluxonium levels in Supplementary Fig. 1 (a). Charge gauge coupling is weighted by $1-\alpha$ and depends on the matrix elements $\omega_{nm}\phi_{nm}$ shown in Supplementary Fig. 1 (b). The matrix elements ϕ_{nm} are largest between adjacent states, so their higher level contributions are suppressed for a sufficiently anharmonic fluxonium system [10]. The same is not true for the

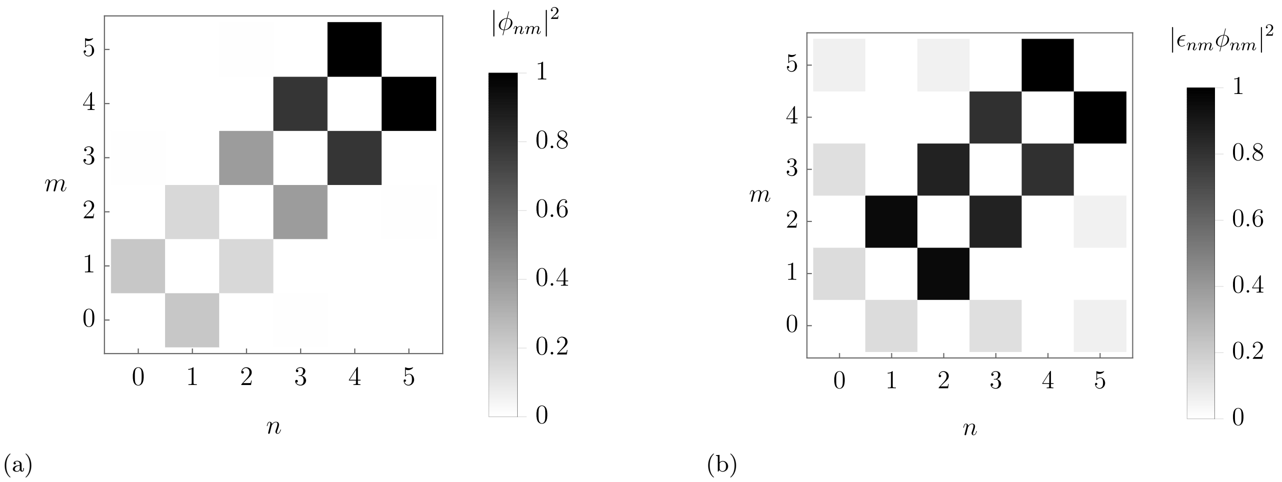

Supplementary Figure 1: **Matrix elements of material canonical operators**. In all plots $E_{\rm l} = 0.33\mu$eV, $E_{\rm J} = 10E_{\rm l} = E_{\rm c}$, and $\phi_{\rm ext} = \pi/2e$. **(a)** Magnitude of dipole matrix elements $|\phi_{nm}|^2$ normalised by the largest element in the array. For sufficiently anharmonic material spectra the contributions of levels $n,\ m > 1$ are negligible **(b)** Magnitude of effective canonical momentum matrix elements $|\epsilon_{nm}\phi_{nm}|^2$ normalised by the largest element in the array. Even for highly anharmonic spectra the contributions of levels $n,\ m > 1$ are non-negligible in general.

matrix elements $\epsilon_{nm}\phi_{nm}$ and this is largely why the charge-gauge QRM is inaccurate even in the regime of small δ where the flux-gauge QRM is relatively accurate in predicting transition energies [10] (Supplementary Note 5).

Let us now consider the three gauges of most importance for our purposes, namely, the flux-gauge, charge gauge and JC-gauge. We begin with the flux-gauge, for which we obtain

$$\kappa_n = g^2 \sum_m \left|\frac{\phi_{nm}}{\varphi}\right|^2 \frac{1}{\epsilon_{nm} - \omega}, \tag{73}$$

$$\chi_n = 2g^2 \sum_m \left|\frac{\phi_{nm}}{\varphi}\right|^2 \frac{\epsilon_{nm}}{\epsilon_{nm}^2 - \omega^2}. \tag{74}$$

For the shifts κ_n contributions from higher material levels $m > 1$ are limited, because the ratios $|\phi_{nm}/\varphi|^2$, $n = 0, 1,\ m > 1$ are generally small [Supplementary Fig. 1 (a)]. Although the χ_n also depend on $|\phi_{nm}/\varphi|^2$ the remaining energy denominator $\epsilon_{nm}/(\epsilon_{nm}^2 - \omega^2)$ is large for levels $m > n = 0,\ 1$ whenever there is a resonance $\epsilon_{mn} \sim \omega$. If δ is large such that there exist higher levels $m > n = 0,\ 1$ for which $\epsilon_m \in [0, \omega]$ it is clear that such resonances can occur, and that they will render higher material levels non-negligible despite the material anharmonicity. The flux-gauge QRM will therefore generally only be accurate for sufficiently small δ and η. We note that this accuracy often includes qualitatively accurate predictions of higher system levels $E_n > E$ for small enough δ and η [10] (see Supplementary Note 5).

In the charge-gauge we obtain

$$\kappa_n = g^2 \sum_m \left|\frac{\phi_{nm}}{\varphi}\right|^2 \frac{1}{\omega\omega_0} \frac{\epsilon_{nm}^2}{\epsilon_{nm} - \omega_0}, \tag{75}$$

$$\chi_n = 2g^2 \sum_m \left|\frac{\phi_{nm}}{\varphi}\right|^2 \frac{1}{\omega\omega_0} \frac{\epsilon_{nm}^3}{\epsilon_{nm}^2 - \omega_0^2}. \tag{76}$$

Like in the flux-gauge resonances in χ_n involving higher material levels can occur and will generally be non-negligible for large δ. The charge-gauge oscillator renormalisation only exacerbates this situation by increasing the oscillator frequency $\omega_0 \geq \omega$. Unlike in the flux-gauge an additional contribution from higher material levels also occurs via the matrix elements $\epsilon_{nm}\phi_{nm}$, For these matrix elements the contribution from higher material levels can be much bigger than that of the lowest two [10] [Supplementary Fig. 1 (b)]. As a result the charge-gauge QRM can be expected to break down even when δ is small and the coupling strength is modest. In fact as explained below the charge-gauge becomes increasingly inaccurate as δ decreases. On the other hand, for large δ the charge-gauge linear coupling term $2E_{\rm c}\xi_0\zeta/e^2$ (ignoring the oscillator renormalisation) has strength $g_0 = g/\delta$ within the two-level truncation. Thus, if δ

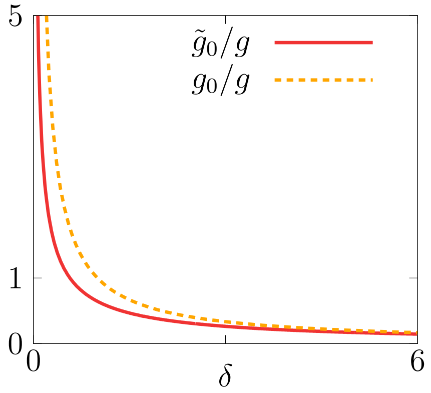

Supplementary Figure 2: **The ratio of charge-gauge QRM and flux-gauge QRM coupling strengths**. In all plots $E_\text{l} = 0.33\mu$eV, $E_\text{J} = 10E_\text{l} = E_\text{c}$, $\phi_\text{ext} = \pi/2e$. The ratio $\tilde{g}_0/g$ of the (renormalised) charge-gauge QRM and flux-gauge QRM coupling strengths is plotted as a function of δ with $\eta = 1$. The effect of the charge-gauge renormalisation of the oscillator frequency is shown by comparison with $g_0/g = 1/\delta$.

is large, e.g., if $\delta = 5$ as considered in the main text and further below (see Supplementary Note 5), then the linear charge-gauge light-matter coupling is much weaker than the corresponding flux-gauge coupling. A more relevant comparison must however account for the renormalisation of the oscillator frequency in the charge-gauge, which is $\omega_0 = \omega\mu_0$ with

$$\mu_0^2 = 1 + \frac{4E_\text{c}}{\omega_\text{m} e^2 \varphi^2}\left[\frac{\eta^2}{\delta}\right]. \tag{77}$$

This renormalisation in turn alters the charge-gauge QRM coupling strength, which becomes $\tilde{g}_0 = g/(\delta\sqrt{\mu_0})$. The ratio $\tilde{g}_0/g$ is shown in Supplementary Fig. 2. Like g_0 the coupling $\tilde{g}_0$ remains relatively weak compared to g for large δ [Supplementary Fig. 2]. Results in the main text and in Supplementary Note 5 confirm that the relative weakness of $\tilde{g}_0$ compared with g allows the charge gauge QRM to (qualitatively) accurately predict the lowest two levels G and E for sufficiently large δ, unless η is very large. In contrast, for large η and small $\delta < 1$ the charge-gauge coupling becomes relatively large compared to the flux-gauge coupling. Combined with the generally non-negligible higher material levels already discussed above, two-level models with $\alpha \sim 0$ can be expected to become rather inaccurate for large η and small δ. Indeed, in agreement with Ref. [10] our results (see Supplementary Note 5) confirm that the performance of the charge-gauge QRM diminishes rapidly with decreasing δ and increasing η even for the first two levels G and E.

Finally we remark on the α_JC-gauge JCM, which symmetrically mixes the flux and charge-gauge QRM couplings. In the JC-gauge we obtain

$$\kappa_n = g^2 \sum_i \left|\frac{\phi_{ni}}{\varphi}\right|^2 \frac{\omega_\text{JC}}{\omega} \frac{(\omega_\text{m} + \epsilon_{ni})^2}{(\epsilon_{ni} - \omega_\text{JC})(\omega_\text{m} + \omega_\text{JC})^2}, \tag{78}$$

$$\chi_n = 2g^2 \sum_i \left|\frac{\phi_{ni}}{\varphi}\right|^2 \frac{\omega_\text{JC}}{\omega} \frac{(\omega_\text{m} + \epsilon_{ni})^2 \epsilon_{ni}}{(\epsilon_{ni}^2 - \omega_\text{JC}^2)(\omega_\text{m} + \omega_\text{JC})^2} \tag{79}$$

where $\omega_\text{m} = \epsilon_{10}$ is the first material transition frequency. By construction, the contributions of the first material level $i = 1$ to the ground shifts κ_0 and χ_0, are zero due to the factor $\omega_\text{m} + \epsilon_{ni}$ in the numerator of both shifts. Thus, in the JC-gauge the bare ground state is only coupled to other levels via counter-rotating contributions involving matrix elements of position and momentum between $|\epsilon_0^\text{JC}\rangle$ and $|\epsilon_i^\text{JC}\rangle$, $i > 1$. These contributions make κ_0 in Supplementary Eq. (78) non-zero in the non-truncated theory. Since κ_0 is the shift of the state $|\epsilon_n^\text{JC}, 0^\text{JC}\rangle$, which is also the ground state of the JC-gauge two-level model, it quantifies the deviation between the ground state of the JC-gauge two-level model and the true ground state $|G\rangle$ of the non-truncated Hamiltonian. In the case of both position and momentum matrix elements the material ground state $|\epsilon_0^\text{JC}\rangle$ is predominantly linked to the first level $|\epsilon_1^\text{JC}\rangle$ (Supplementary Fig. 1). Matrix elements involving $|\epsilon_0^\text{JC}\rangle$ and higher levels are smaller especially in the case of the flux operator ϕ. As a result κ_0 is small in the JC-gauge, which explains why the JC-gauge two-level model gives a good representation of the

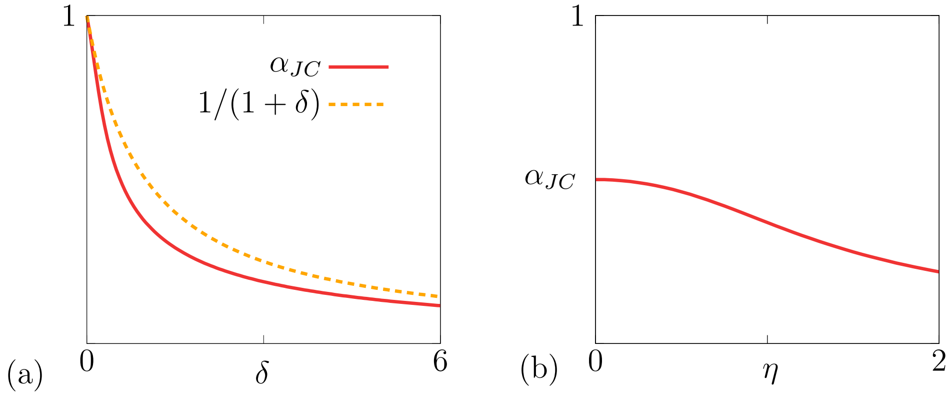

Supplementary Figure 3: **The $\alpha_{\rm JC}$-gauge parameter as a function of coupling and detuning**. In all plots $E_{\rm l} = 0.33\mu$eV, $E_{\rm J} = 10E_{\rm l} = E_{\rm c}$, $\phi_{\rm ext} = \pi/2e$. **(a)** $\alpha_{\rm JC}$ and $1/(1+\delta)$ as a function of δ with $\eta = 1$. **(b)** $\alpha_{\rm JC}$ as a function of η with $\delta = 1$.

ground state. Indeed our results confirm that the JC gauge two-level model actually always outperforms the available QRMs in predicting the ground state and its energy.

To understand the accuracy of the JC-gauge two-level model more generally we consider how $\alpha_{\rm JC}$ varies with δ and η. This is shown in Supplementary Fig. 3. The effect of the renormalisation of the oscillator frequency is shown by comparison with $\omega_{\rm m}/(\omega+\omega_{\rm m}) = 1/(1+\delta)$ which equals $\alpha_{\rm JC}$ if $\omega_{\rm JC}$ is approximated as ω. As δ increases $\alpha_{\rm JC}$ decays quite rapidly from 1 towards 0 indicating that the JC-gauge becomes predominantly charge-like for even fairly small δ. For large δ both the flux and charge-gauge QRMs are inaccurate for levels $E_n > E$, and the JC-gauge is therefore also inaccurate for these levels. More surprising is the inaccuracy of the JC-gauge for levels $E_n > E$ when δ is small. For example, when $\delta = 1/5$ the flux-gauge QRM is relatively accurate (see Supplementary Note 5) and for this value of δ we obtain $\alpha_{\rm JC} \sim 0.8$ when $\eta = 1$ indicating a predominantly flux-like coupling within the JC-gauge. For $\delta = 1/5$ the JC-gauge two-level model is nevertheless relatively inaccurate (compared with the flux-gauge QRM) in predicting levels $E_n > E$ for sufficiently large couplings (see Supplementary Note 5). This can only be attributed to the quite severe breakdown of the charge-gauge QRM for small δ combined with the decrease in $\alpha_{\rm JC}$ towards the charge-gauge value 0, as the coupling η increases [Supplementary Fig. 3 (b)].

Supplementary Note 5: Further anaysis; alternative parameter regimes

Here we provide further analysis of two-level models via comparison with exact predictions. Our findings are consistent with the analysis of Supplementary Note 4 above. We divide this part into three sections corresponding to three values of the detuning $\delta = 1/5,\ 1,\ 5$. For each value of δ we consider variations in the remaining parameters $\eta,\ \phi_{\rm ext},\ \alpha$. For large detuning $\delta = 5$ two-level models tend to be inaccurate in predicting dressed energies $E_n > E$ with E the first excited level. The charge gauge QRM is more accurate than the flux gauge QRM for the first two levels while the JC-gauge is the most accurate two-level model. As δ decreases the charge gauge QRM becomes less accurate and the flux-gauge QRM more accurate. The JC-gauge two-level model typically remains the most accurate for the first two system levels, but unlike the charge-gauge QRM and JC-gauge two-level model the flux gauge QRM is able to give qualitative agreement with the non-truncated model for levels $E_n > E$ provided δ and η are sufficiently small.

1. $\delta = 5$

We begin with the most experimentally relevant regime presently, $\delta = 5$. We show in Supplementary Fig. 4 how the first two dressed energies behave as $\phi_{\rm ext}$ is varied within the deep-strong coupling regime $\eta = 1.5$. The JC-gauge two-level model provides reasonable qualitative agreement with the exact energies while the flux and charge-gauge QRMs are significantly less accurate. The flux and charge-gauge JCMs are also inaccurate as expected.

Next we restrict our attention to the maximal frustration $\phi_{\rm ext} = \pi/2e$ point and consider how predictions vary with α while other parameters are held fixed. Supplementary Fig. 5 shows how the dressed energies of the general α-gauge two-level model varies with α when $\eta = 1$. All two-level models become inaccurate for dressed levels $E_n > E$. For the specified parameters two-level models with α near to $\alpha_{\rm JC} = 0.132$ are accurate in predicting the first two energy values.

Supplementary Fig. 6 shows how the ground and excited state state fidelities $F^\alpha_{g,e}$ vary with α for various couplings. For sufficiently small η the JC-gauge two-level model is always close to the optimal two-level model for representing the ground state. For larger η degeneracy points of the first two-levels occur for certain values of α. For such α a transfer of population within the exact ground state $|G\rangle$ from the ground state $|G^\alpha_2\rangle$ into the excited state $|E^\alpha_2\rangle$ occurs, such that the state $|E^\alpha_2\rangle$ becomes a better representation of the true ground state $|G\rangle$. In fact for large enough η there exists a value $\alpha_{\rm opt}$ that through $|E_2^{\alpha_{\rm opt}}\rangle$ gives the best possible representation of $|G\rangle$, i.e., is such that $|\langle E_2^{\alpha_{\rm opt}}|G\rangle|^2 \geq F^\alpha_g,\ \forall\alpha$.

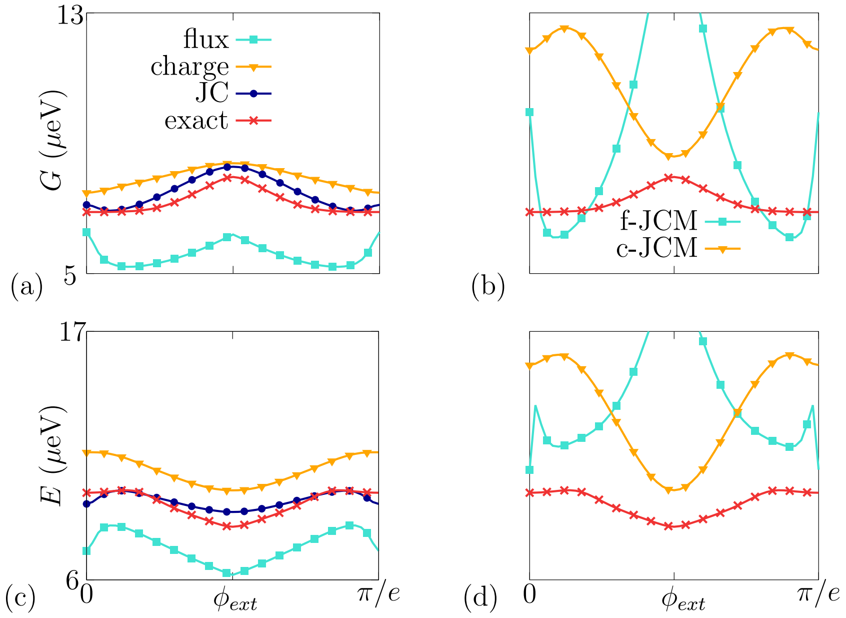

Supplementary Figure 4: **Lowest energy levels as functions of external flux**. In all plots $E_{\rm l} = 0.33\mu$eV, $E_{\rm J} = 10E_{\rm l} = E_{\rm c}$, $\delta = 5$ and $\eta = 1.5$. **(a)** The ground energy is plotted with $\phi_{\rm ext}$ for the flux-gauge and charge-gauge QRMs, for the JC-gauge two-level model and for the exact model (solid red). **(b)** For the same range as (a) the ground energy is plotted with $\phi_{\rm ext}$ for the flux-gauge and charge-gauge JCMs, and for the exact model. **(c)** Same as (a) for the first excited energy. **(d)** Same as (b) for the first excited energy.

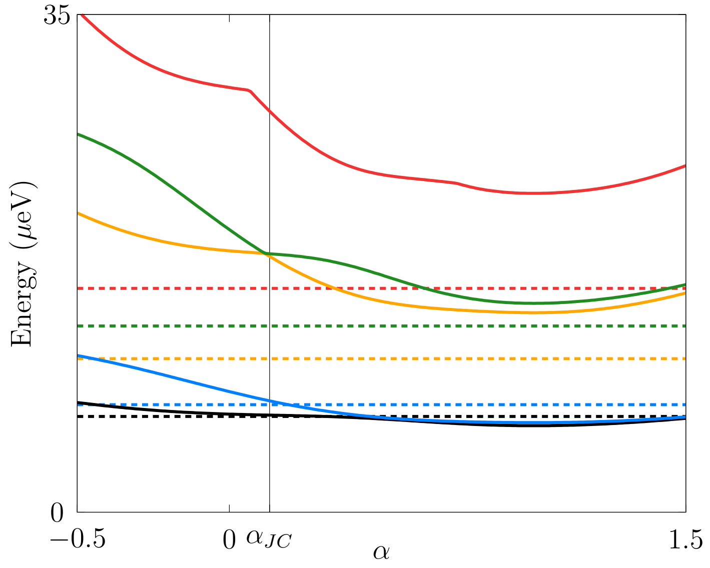

Supplementary Figure 5: **Lowest energy levels as functions of the gauge parameter**. In all plots $E_\mathrm{l} = 0.33\mu$eV, $E_\mathrm{J} = 10E_\mathrm{l} = E_\mathrm{c}$, $\delta = 5$, $\phi_\mathrm{ext} = \pi/2e$ and $\eta = 1$. The first five Hamiltonian energies are plotted as functions of α. The dashed lines give the α-independent exact energies, while the solid curves give the corresponding energies found within the two-level truncation. The vertical line specifies the value α_JC.

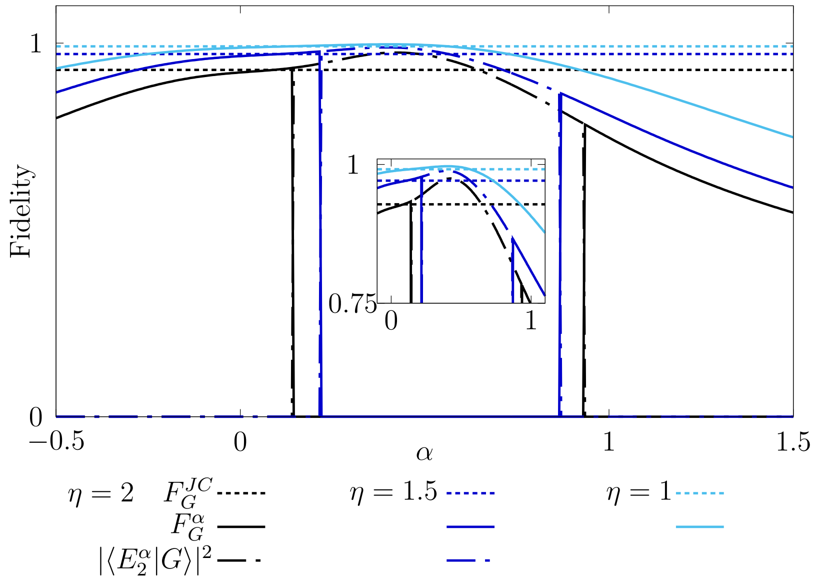

Supplementary Figure 6: **Fidelities of the lowest eigenstates as functions of the gauge parameter**. $E_\mathrm{l} = 0.33\mu$eV, $E_\mathrm{J} = 10E_\mathrm{l} = E_\mathrm{c}$, $\delta = 5$ and $\phi_\mathrm{ext} = \pi/2e$. The ground state fidelity of two-level model states $|G_2^\alpha\rangle$ and $|E_2^\alpha\rangle$ in the ground state $|G\rangle$ are plotted as functions of α for $\eta = 1,\ 1.5,\ 2$ (solid lines). The straight dashed lines show the values of F_G^JC for the same three coupling strengths. The dash-dotted lines show the overlap $|\langle E_2^\alpha|G\rangle|^2$ as a function of α for $\eta = 1.5,\ 2$.

2. $\delta = 1$

Next we consider the case of resonance $\delta = 1$. Here for the first two levels the JC-gauge two-level model again provides energies closest to the exact energies. The JC-gauge again also provides the best representation of the ground state (Supplementary Fig. 7). The flux-gauge QRM becomes more accurate and is typically more accurate than the charge-gauge QRM (Supplementary Fig. 8). For $\eta > 0.4$ the excited state of the flux-gauge QRM has larger overlap with the exact excited state than the JC-gauge JCM. Two-level models remain largely inaccurate for levels $E_n > E$

in this regime of detuning, although the flux-gauge QRM accurately predicts certain energies for levels $E_n > E$ e.g. $n = 3,\ 5,\ 8$ as shown in Supplementary Fig. 9 for $\eta = 1$.

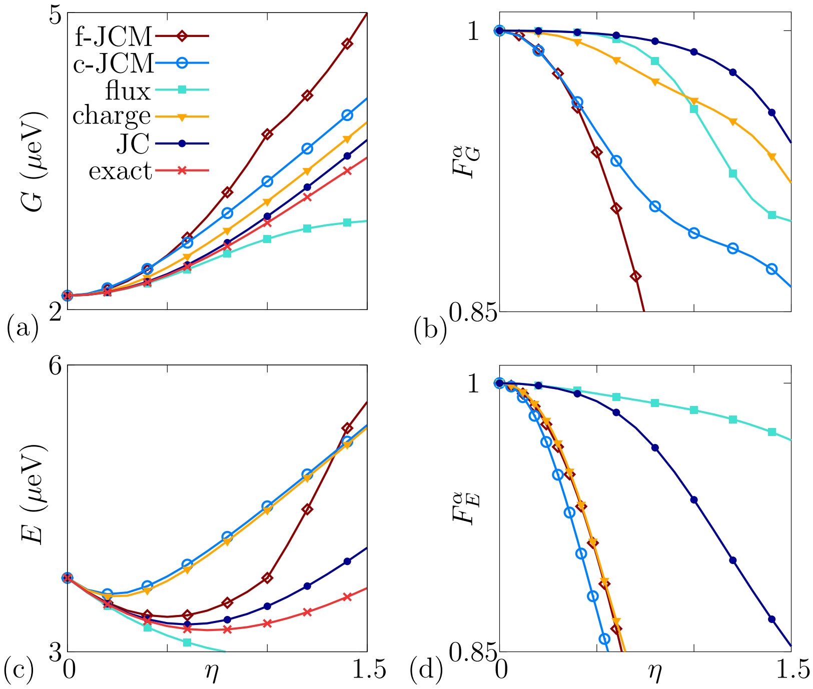

Supplementary Figure 7: **Lowest energies as functions of coupling strength**. In all plots $E_{\rm l} = 0.33\mu$eV, $E_{\rm J} = 10E_{\rm l} = E_{\rm c}$, $\delta = 1$ and $\phi_{\rm ext} = \pi/2e$. **(a)** The ground energy is plotted with η for the flux-gauge and charge-gauge QRMs, for the JC-gauge two-level model, for the exact model, and for the flux and charge-gauge JCMs obtained via the RWA. **(b)** The ground state fidelity F_G^α is plotted with η for the flux and charge-gauge QRMs, for the JC-gauge two-level model and for the flux and charge-gauge JCMs. **(c)** Same as (a) for the first excited energy. **(d)** Same as (b) for the first excited state.

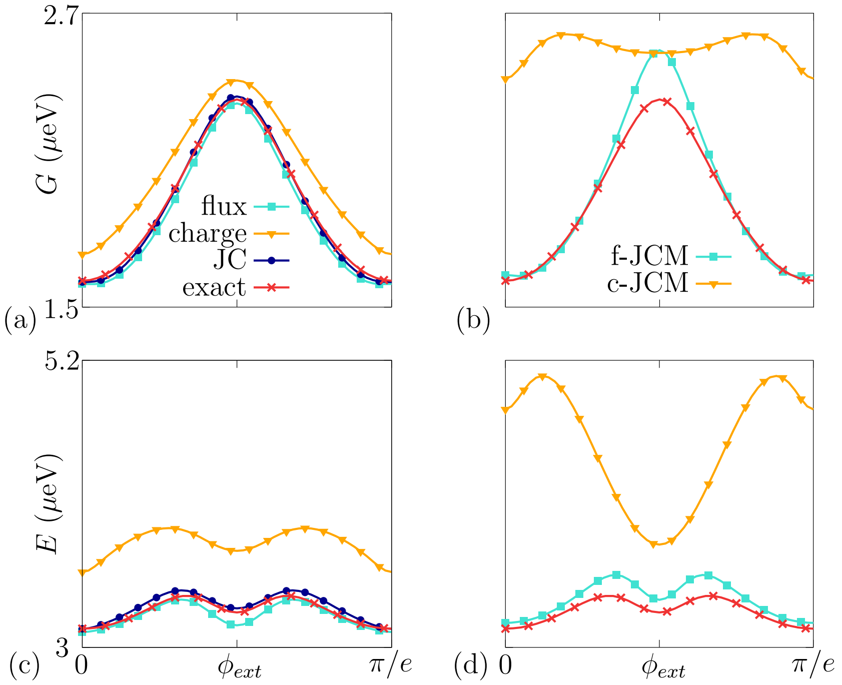

Supplementary Figure 8: **Lowest energies as functions of external flux**. In all plots $E_l = 0.33\mu$eV, $E_J = 10E_l = E_c$, $\delta = 1$ and $\eta = 1/2$. **(a)** The ground energy is plotted with ϕ_{ext} for the flux-gauge and charge-gauge QRMs, for the JC-gauge two-level model and for the exact model. **(b)** For the same range as (a) the ground energy is plotted with ϕ_{ext} for the flux-gauge and charge-gauge JCMs, and for the exact model. **(c)** Same as (a) for the first excited energy. **(d)** Same as (b) for the first excited energy.

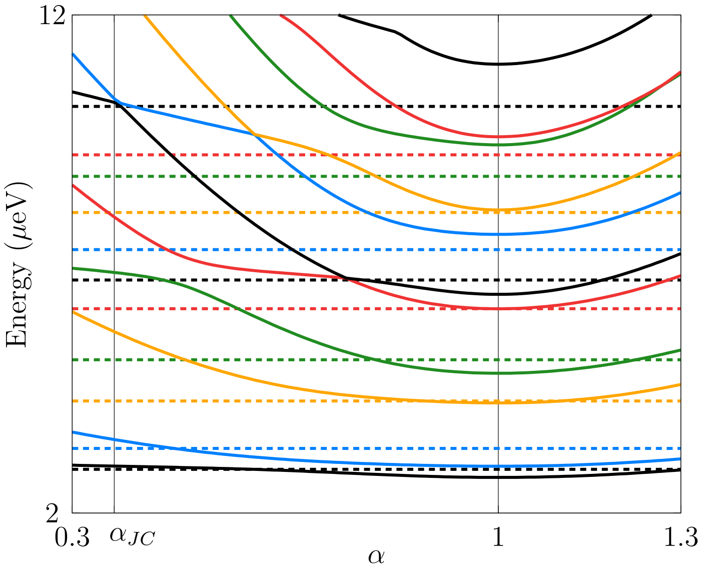

Supplementary Figure 9: **Lowest energies as functions of the gauge parameter**. $E_l = 0.33\mu$eV, $E_J = 10E_l = E_c$, $\delta = 1$, $\phi_{ext} = \pi/2e$ and $\eta = 1$. The first 11 Hamiltonian energies are plotted as functions of α. The dashed lines give the α-independent exact energies, while the solid curves give the corresponding energies found within the two-level truncation. The vertical lines specifiy the value α_{JC} and the flux-gauge $\alpha = 1$ respectively.

3. $\delta = 1/5$

Next we consider the regime $\omega_0 > \omega$ by letting $\delta = 1/5$. Here the JC-gauge again provides the best representation of the ground state and for the first two levels again provides energies closest to the exact energies (Supplementary Fig. 10). The flux-gauge QRM becomes yet more accurate while the charge-gauge QRM becomes yet more inaccurate (Supplementary Fig. 11). As in the case of the charge-gauge QRM in the regime $\delta = 5$ the RWA is seen to incur very little error when applied to the flux-gauge QRM in the regime $\delta = 1/5$. The flux-gauge JCM therefore occasionally outperforms the charge-gauge QRM (Supplementary Fig. 11). The flux-gauge QRM becomes much more accurate at predicting energy values for levels $E_n > E$ as shown in Supplementary Fig. 12. Again all two-level models breakdown for these levels when the coupling is sufficiently large.

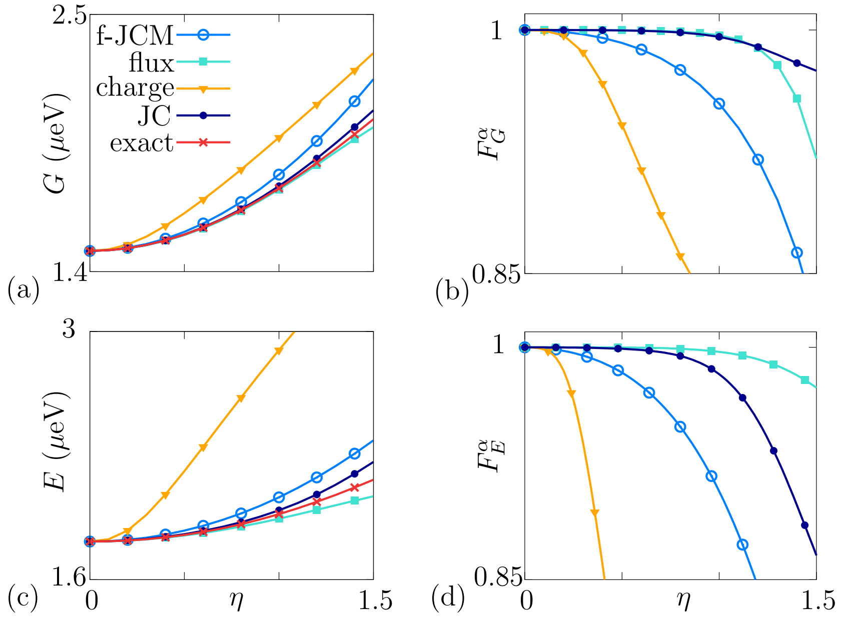

Supplementary Figure 10: **Lowest energies as functions of coupling strength**. In all plots $E_\mathrm{l} = 0.33\mu\mathrm{eV}$, $E_\mathrm{J} = 10E_\mathrm{l} = E_\mathrm{c}$, $\delta = 1/5$ and $\phi_\mathrm{ext} = \pi/2e$. **(a)** The ground energy is plotted with η for the flux-gauge and charge-gauge QRMs, for the JC-gauge two-level model, for the exact model, and for the charge-gauge JCM obtained via the RWA. **(b)** The ground state fidelity F_G^α is plotted with η for the flux-gauge $\alpha = 1$ and charge-gauge $\alpha = 0$ QRMs, for the JC-gauge and for charge-gauge JCM. **(c)** Same as (a) for the first excited energy. **(d)** Same as (b) for the first excited state.

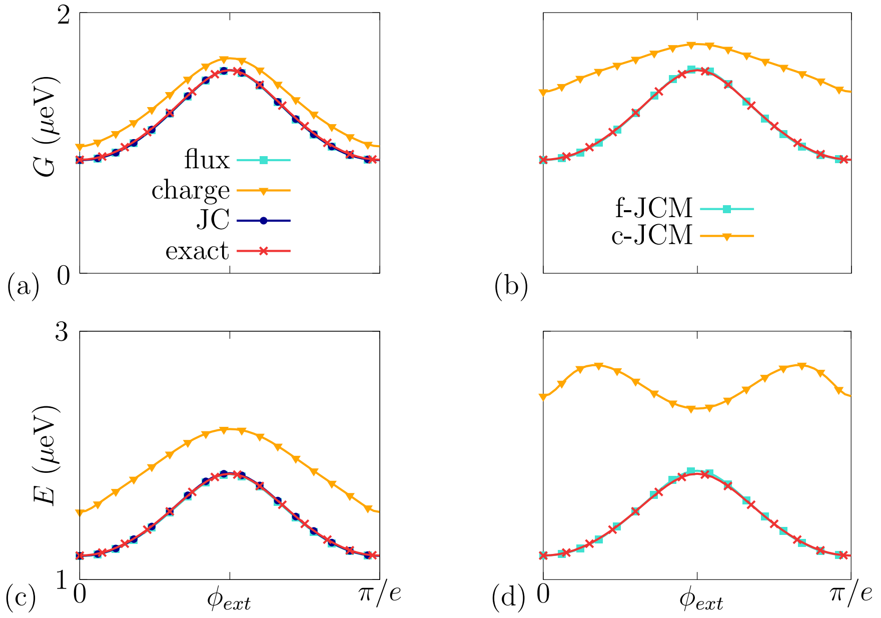

Supplementary Figure 11: **Lowest energies as functions of external flux**. In all plots $E_{\rm l} = 0.33\mu$eV, $E_{\rm J} = 10E_{\rm l} = E_{\rm c}$, $\delta = 1/5$ and $\eta = 1/2$. **(a)** The ground energy is plotted with $\phi_{\rm ext}$ for the flux-gauge and charge-gauge QRMs, for the JC-gauge two-level model and for the exact model (solid red). **(b)** For the same range as (a) the ground energy is plotted with $\phi_{\rm ext}$ for the flux-gauge and charge-gauge JCMs, and for the exact model. **(c)** Same as (a) for the first excited energy. **(d)** Same as (b) for the first excited energy.

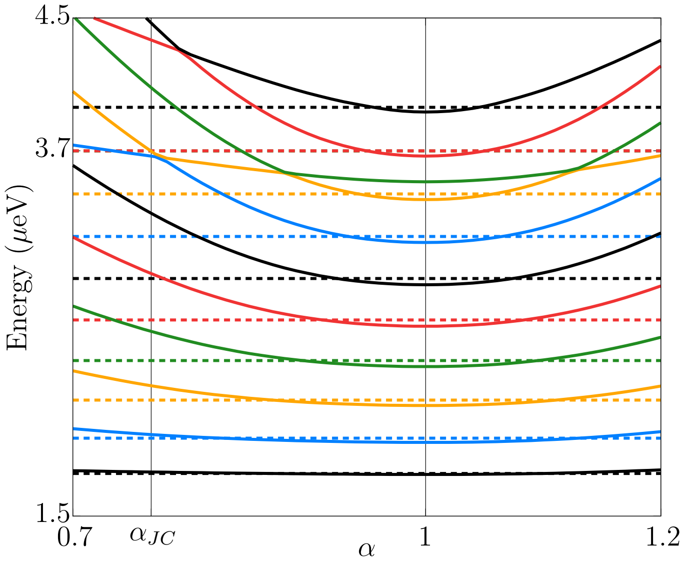

Supplementary Figure 12: **Lowest energies as functions of the gauge parameter**. $E_{\rm l} = 0.33\mu$eV, $E_{\rm J} = 10E_{\rm l} = E_{\rm c}$, $\delta = 1/5$, $\phi_{\rm ext} = \pi/2e$ and $\eta = 1$. The first 11 Hamiltonian energies are plotted as functions of α. The dashed lines give the α-independent exact energies, while the solid curves give the corresponding energies found within the two-level truncation. The vertical lines specifiy the value $\alpha_{\rm JC}$ and the flux-gauge $\alpha = 1$ respectively. The exact energies for levels 9 and 10 are nearly equal at 3.6995μeV and 3.6997μeV respectively.

Supplementary Note 6: Photon number averages

As an example of an observable different from the energy we consider here photon number. Each different gauge α has two associated photon number operators, $a_\alpha^\dagger a_\alpha$ and $c_\alpha^\dagger c_\alpha$. The number operator $a_\alpha^\dagger a_\alpha$ has associated energy $H_{\rm c}^\alpha = \omega(a_\alpha^\dagger a_\alpha + 1/2)$, whereas the number operator $c_\alpha^\dagger c_\alpha$ implicitly includes the oscillator self-energy contribution $E_{\rm c}(1-\alpha)^2\zeta^2/e^2$ such that the associated energy is $H_{\rm c}^\alpha + E_{\rm c}(1-\alpha)^2\zeta^2/e^2 = \omega_\alpha(c_\alpha^\dagger c_\alpha + 1/2)$ where $\omega_\alpha^2 = \omega^2 + E_{\rm c}(1-\alpha)^2 C\zeta^2/e^2$. If and only if $\alpha = 1$ does one obtain $c_\alpha^\dagger c_\alpha = a_\alpha^\dagger a_\alpha$. We focus on the renormalised number operator $c_\alpha^\dagger c_\alpha$, which can be expressed in terms of operators associated with any other gauge $\alpha' \neq \alpha$ by using the unitary relation between c_α and a_α and then using the unitary relation between a_α and $a_{\alpha'}$. Explicitly we have

$$n_\alpha = c_\alpha^\dagger c_\alpha = \frac{1}{2}\left(\frac{\omega}{\omega_\alpha} + \frac{\omega_\alpha}{\omega}\right) a_\alpha^\dagger a_\alpha + \frac{1}{4}\left(\frac{\omega}{\omega_\alpha} - \frac{\omega_\alpha}{\omega}\right)(a_\alpha^{\dagger\,2} + a_\alpha^2) + \frac{1}{4}\left(\frac{\omega}{\omega_\alpha} + \frac{\omega_\alpha}{\omega} - 2\right), \tag{80}$$

and

$$a_\alpha = a_{\alpha'} + \frac{\eta}{\varphi}(\alpha' - \alpha)\phi. \tag{81}$$

Upon substitution of Supplementary Eq. (81) into the right-hand-side of Supplementary Eq. (80) one obtains $n_\alpha = n_\alpha(\mathbf{y}_{\alpha'})$ expressed as a function of α'-gauge ladder operators and ϕ. The expression includes terms quadratic in ϕ implying that, like the Hamiltonian, there are at least two non-equivalent ways of defining n_α within the α'-gauge two-level model, because $P^{\alpha'}\phi^2 P^{\alpha'} \neq (P^{\alpha'}\phi P^{\alpha'})^2$ (see Supplementary Note 2). The two possible definitions of n_α in the α'-gauge two-level model are given by the left and right-hand-sides of the inequality

$$P^{\alpha'} n_\alpha(\mathbf{y}_{\alpha'}) P^{\alpha'} \neq n_\alpha(P^{\alpha'}\mathbf{y}_{\alpha'}P^{\alpha'}). \tag{82}$$

Supplementary Fig. 13 shows the averages $\langle c_1^\dagger c_1\rangle_G$ and $\langle c_1^\dagger c_1\rangle_E$ in the ground and first energy states $|G\rangle$ and $|E\rangle$ found using various two-level models and the exact model. The predictions of two-level models in gauges other than the flux-gauge are found using the definition given by the right-hand-side of the inequality (82). The JC-gauge two-level model is more accurate than the flux-gauge QRM, the flux and charge-gauge JCMs, and is comparable to the charge-gauge QRM.

As a second example we consider the JC-gauge photon number operator $c_{\rm JC}^\dagger c_{\rm JC}$. In the ground state of the JC-gauge two-level model the average of this observable is zero for all coupling strengths. Thus, the exact average should be approximately zero for coupling strengths for which higher material levels can be neglected within the JC-gauge. Supplementary Fig. 14 shows that the exact average is zero for sufficiently small coupling, but begins to grow for larger coupling strengths, which gives an indication of the relative validity of the JC-gauge two-level truncation. The flux-gauge QRM prediction for this observable is given for definitions provided by both the right and left-hand-sides of the inequality (82). These definitions are labelled type 1 and type 2 respectively. In both cases the flux-gauge QRM overestimates the average and is less accurate than the JC-gauge two-level model.

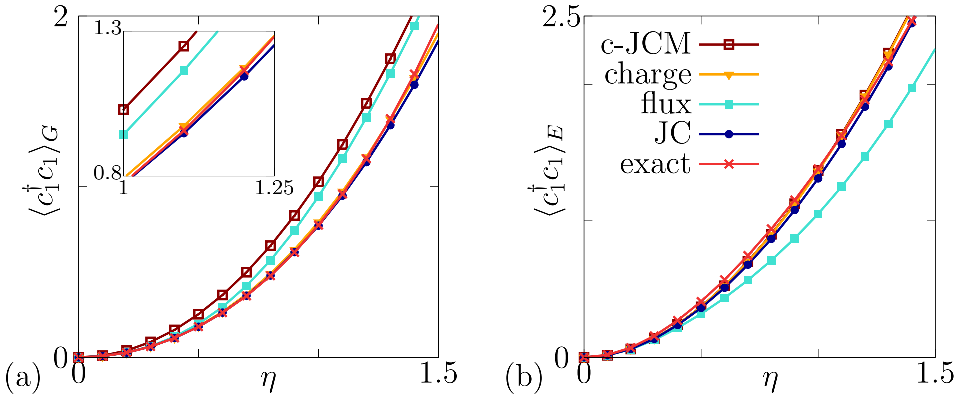

Supplementary Figure 13: **Predictions of the number of flux-gauge photons as functions of coupling strength**. In all plots $E_{\rm l} = 0.33\mu$eV, $E_{\rm J} = 10E_{\rm l} = E_{\rm c}$, $\delta = 5$ and $\phi_{\rm ext} = \pi/2e$. **(a)**. The ground state average flux-gauge photon number is plotted with coupling η, for the flux-gauge and charge-gauge QRMs, for the JC-gauge two-level model, the exact theory, and the charge-gauge JCM (c-JCM). The flux-gauge JCM is inaccurate in the regime considered and is not shown. **(b)** Same as (a) for the first excited state average. In both graphs the plots corresponding to two-level models in gauges other than the flux gauge are found using the definition on the right-hand-side of inequality (82). The charge-gauge QRM, JC-gauge two-level model, and the exact number prediction are very close together, and in the case of the excited state the charge-gauge JCM is also accurate in this regime.

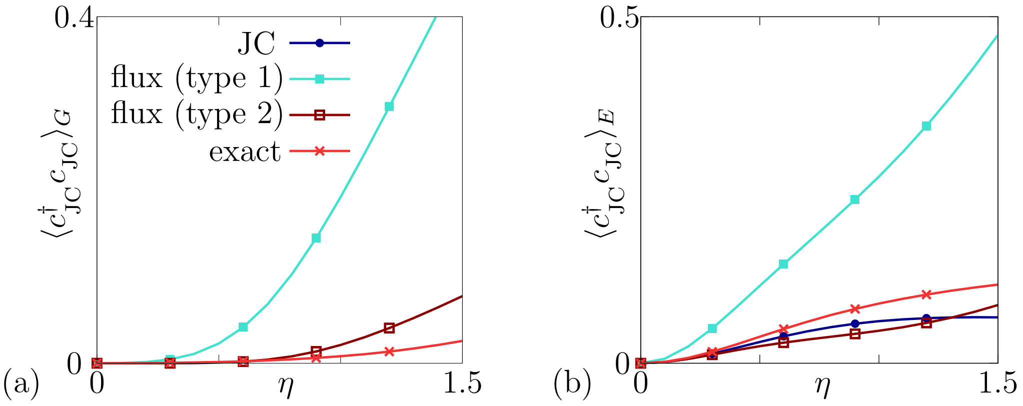

Supplementary Figure 14: **Predictions of the number of JC-gauge photons as functions of coupling strength**. In all plots $E_{\rm l} = 0.33\mu$eV, $E_{\rm J} = 10E_{\rm l} = E_{\rm c}$, $\delta = 5$ and $\phi_{\rm ext} = \pi/2e$. **(a)** The ground state average JC-gauge photon number is plotted with coupling η, for the flux-gauge and the exact theory. The exact result remains approximately zero well into the ultrastrong regime indicating that the JC-gauge two-level model remains accurate. **(b)** Same as (a) for the first excited state average. The JC-gauge two-level model prediction is not identically zero in the excited state and has been included. In both graphs the flux-gauge QRM plots are given for definitions provided by both the right and left-hand-sides of the inequality (82), which are labelled type 1 and type 2 respectively. In both cases the flux-gauge QRM is less accurate than the JC-gauge two-level model in the ultrastrong-coupling regime.

[1] Woolley R. G. Charged particles, gauge invariance, and molecular electrodynamics. *International Journal of Quantum Chemistry* **74**, 531–545 (1999). URL `https://onlinelibrary.wiley.com/doi/abs/10.1002/%28SICI%291097-461X%281999%2974%3A5%3C531%3A%3AAID-QUA9%3E3.0.CO%3B2-H`.

[2] Stokes, A. Noncovariant gauge fixing in the quantum Dirac field theory of atoms and molecules. *Physical Review A* **86**, 012511 (2012). URL `https://link.aps.org/doi/10.1103/PhysRevA.86.012511`.

[3] Cohen-Tannoudji, C., Dupont-Roc, J. & Grynberg, G. *Photons and Atoms: Introduction to Quantum Electrodynamics* (Wiley-VCH, Weinheim, 1997), 1st edn.

[4] Dirac, P. A. M. *Lectures on Quantum Mechanics* (Dover Publications Inc., Mineola, NY, 2003).

[5] Barton, G. Frequency shifts near an interface: inadequacy of two-level atomic models. *Journal of Physics B: Atomic and Molecular Physics* **7**, 2134 (1974). URL http://stacks.iop.org/0022-3700/7/i=16/a=012.

[6] Craig, D. P. & Thirunamachandran, T. *Molecular Quantum Electrodynamics: An Introduction to Radiation-molecule Interactions* (Courier Corporation, 1998). Google-Books-ID: rpbdozIZt3sC.

[7] Stokes, A. & Nazir, A. A master equation for strongly interacting dipoles. *New Journal of Physics* **20**, 043022 (2018). URL http://stacks.iop.org/1367-2630/20/i=4/a=043022.

[8] Manucharyan, V. E., Baksic, A. & Ciuti, C. Resilience of the quantum Rabi model in circuit QED. *Journal of Physics A: Mathematical and Theoretical* **50**, 294001 (2017). URL http://stacks.iop.org/1751-8121/50/i=29/a=294001.

[9] Zhu, G., Ferguson, D. G., Manucharyan, V. E. & Koch, J. Circuit QED with fluxonium qubits: Theory of the dispersive regime. *Physical Review B* **87**, 024510 (2013). URL https://link.aps.org/doi/10.1103/PhysRevB.87.024510.

[10] De Bernardis, D., Pilar, P., Jaako, T., De Liberato, S. & Rabl, P. Breakdown of gauge invariance in ultrastrong-coupling cavity QED. *Phys. Rev. A* **98**, 053819 (2018). URL https://link.aps.org/doi/10.1103/PhysRevA.98.053819.
